# A Molecular Recruitment Colocalization Platform for Visualizing Multi‐Protein Interactions and Engineering Biomolecular Condensates in Living Cells

**DOI:** 10.1002/advs.202505455

**Published:** 2025-07-22

**Authors:** Lei Peng, Yu Hou, Yan Zhao, Jing‐Ya Tang, Liu Song, Bo Peng, Min Li, Dian‐Bing Wang, Xian‐En Zhang

**Affiliations:** ^1^ Institute of Biophysics Chinese Academy of Sciences Beijing 100101 China; ^2^ College of Life Sciences Guizhou Normal University Guiyang 550025 China; ^3^ Faculty of Synthetic Biology Shenzhen University of Advanced Technology Shenzhen 518107 China; ^4^ State Key Laboratory of Hybrid Rice, Hunan Hybrid Rice Research Center Hunan Academy of Agricultural Sciences Changsha 410125 China

**Keywords:** colocalization, condensates, live cell imaging, multi‐protein interactions, phase separation, protein binding ability

## Abstract

Visualizing dynamic protein–protein interactions (PPIs) and phase‐separated biomolecular condensates in live cells is crucial for understanding cellular processes. Here, a method is introduced for analyzing both PPIs and condensates, termed molecular recruitment colocalization (MRC), which localizes interacting partners and generates fluorescent signals at designated genomic loci. MRC enables the simultaneous visualization of multi‐protein interactions and the preliminary estimation of binding affinities during PPIs. Through MRC, the study can quickly discover and validate new proteins or structural domains capable of forming condensates and can also integrate PPIs to study condensates. Furthermore, the versatility of MRC is demonstrated in phase‐separated condensates research: programming phase separation sites to study the interactions and behaviors between different condensates, recruiting target proteins into programmable condensates, obtaining the properties of diverse engineered condensates, and probing the physical properties of condensates within live cells. Therefore, MRC can become a versatile platform for studying both PPIs and biomolecular condensates.

## Graphical abstract

MRC is a live‐cell method enabling visualization of protein–protein interactions and biomolecular condensates. It supports multi‐protein colocalization, binding analysis, and programmable condensate manipulation, providing a versatile platform for studying dynamic cellular organization.

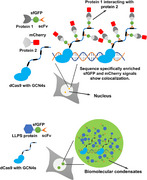

## 1 Introduction

Protein–protein interactions (PPIs) play a key role in a wide range of biological processes. Various methods have been developed to visualize PPIs in live cells, including fluorescence resonance energy transfer (FRET),^[^ ^1^ ^]^ bioluminescence resonance energy transfer (BRET),^[^ ^2^, ^3^ ^]^ and bimolecular fluorescence complementation (BiFC).^[^ ^4^, ^5^, ^6^, ^7^ ^]^ Colocalization imaging methods include the single‐molecule protein proximity index (smPPI),^[^ ^8^ ^]^ 3D colocalization,^[^ ^9^ ^]^ and 3D dual‐particle tracking.^[^ ^10^ ^]^ Although these methods have been widely applied, they have inherent limitations. FRET is susceptible to autofluorescence and background light,^[^ ^11^, ^12^ ^]^ whereas BiFC may yield false positives owing to the spontaneous complementation of overexpressed protein fragments, and slow chromophore maturation and irreversibility of complementary fluorescent proteins hinders the visualization of transient interactions.^[^ ^13^ ^]^ Colocalization imaging requires intricate super‐resolution microscopy for high spatial correlation.^[^ ^14^, ^15^ ^]^ Furthermore, due to these limitations, these methods face challenges in the study of multi‐protein complexes in live cells,^[^ ^16^ ^]^ though there are already a few prelimilary investigations.^[^ ^17^, ^18^, ^19^ ^]^

Evidence is now mounting that liquid–liquid phase separation (LLPS) underlies the formation of membraneless compartments in cells^[^ ^20^, ^21^, ^22^, ^23^, ^24^, ^25^ ^]^ and LLPS span the cell, from the nucleus to cytoplasm and cellular membranes, exemplified by nucleoli, nuclear speckles, cajal bodies, stress granules, P‐bodies, and post‐synaptic density zones.^[^ ^26^ ^]^ In addition to the in vitro methods used in studies on phase‐separated condensates, current methods for intracellular applications mainly include fluorescence recovery after photobleaching, fluorescence correlation spectroscopy, and live cell optical morphology imaging.^[^ ^27^, ^28^ ^]^ These methods are simple and reliable, but they would not be applicable if the target proteins did not form condensates within cells or did not adopt the typical liquid condensate morphology in cellular contexts. Distinguishing the phase separation and exploring the properties of condensates in vivo remain challenging.^[^ ^27^, ^28^ ^]^

Previous studies introduced the Fluorescent‐Three‐Hybrid (F3H) method, which recruits proteins to specific genomic loci using a Lac operator array and visualizes protein interactions through colocalization.^[^ ^29^ ^]^ However, compared to BiFC and FRET, its application has been less widespread. F3H is primarily used to validate pairwise protein interactions but has not been extended to analyze interactions within multiprotein complexes. Additionally, this method does not assess protein binding affinity quantitatively, and subsequent studies have not further optimized or expanded its application.

Recently, optogenetic tools have been developed to locally induce condensation in live cells through controlled multivalent changes, which are useful for controlling phase transitions within living cells.^[^ ^30^, ^31^, ^32^ ^]^ However, these optogenetic tools have limitations when studying in vivo phase separation. The oligomerization of optogenetic proteins increases system multivalency, thereby affecting the accurate assessment of protein phase separation properties.^[^ ^27^, ^28^ ^]^ Therefore, the development of precise, robust, modular, programmable, and adaptable tools for generating different types of condensates within live cells is essential.

In this study, we introduced a strategy termed the molecular recruitment colocalization system (MRC), which is a dual‐function method capable of visualizing and validating PPIs of multi‐protein complexes and biomolecular condensates within live cells. This system enables us to visualize and effectively validate various protein interactions in live cells, and in particular, simultaneously observe interactions between multiple protein complexes and initially assess their binding ability among the proteins. Using MRC, we compared the interaction‐binding capabilities of different G‐protein complex members. Additionally, MRC enables the rapid screening of proteins or domains with condensate formation capabilities. We demonstrated that multiple proteins containing PDZ domains can form phase‐separated condensates. Moreover, owing to its dual functions, MRC exhibits versatility in the exploration of cellular events, which include but not limited to: examining how the interactions between multiple protein complexes promote condensates formation, as we observed that the interaction between PSD95 and NOS1 can specifically induce phase separation at the N‐terminus of NOS1; exploring the interactions of different condensates by combining MoonTag protocol to create local phase separation of different proteins in the same area; selectively inducing phase separation in condensates containing any protein of interest using optogenetic tools in live cells; and tracking the dynamics of phase‐separating proteins within condensates at the single‐molecule level by incorporating quantum dots as fluorescent probes.

## 2 Results

### 2.1 Visualization of PPIs in Live Cells Through Molecular Recruitment Colocalization Analysis

**Figure 1:**
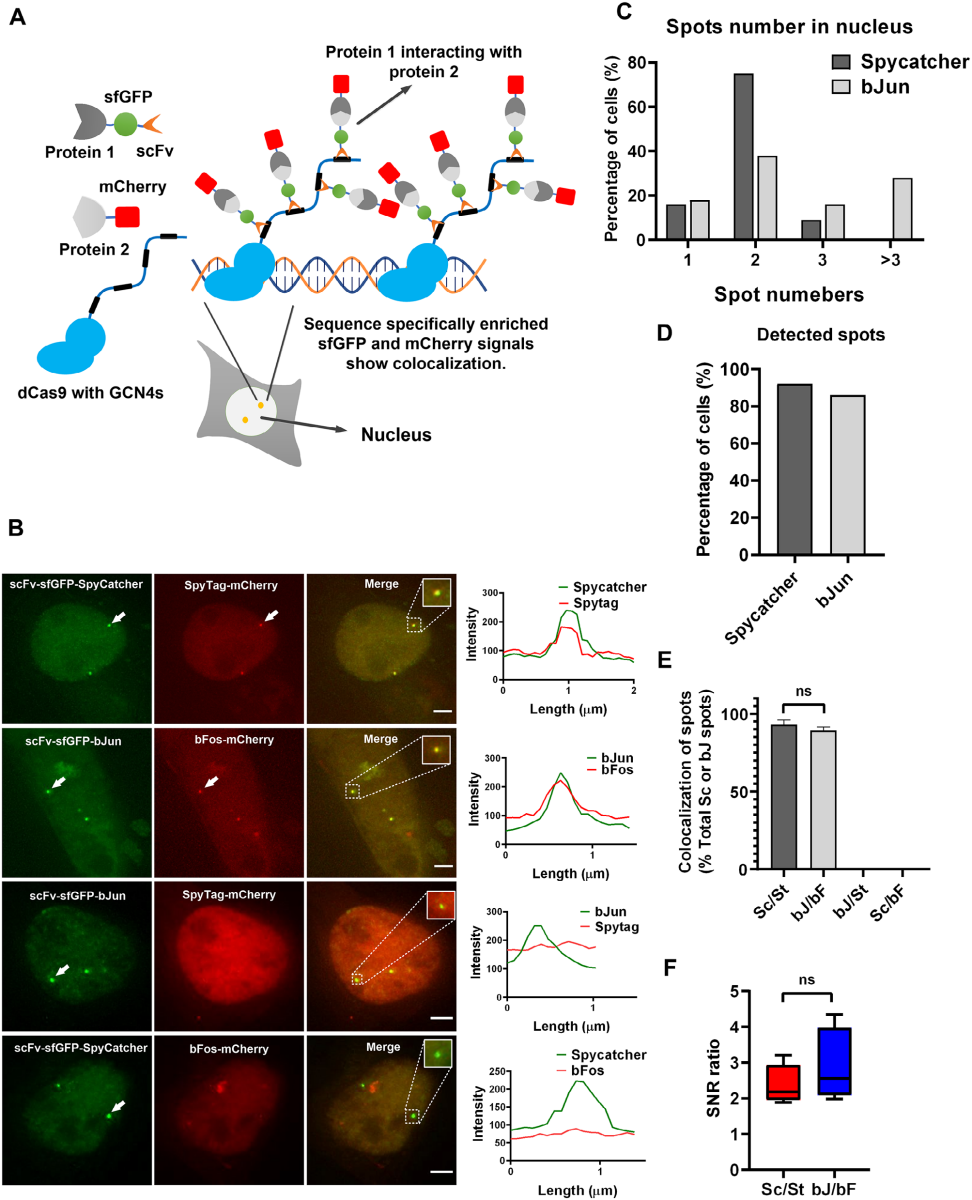
Visualization of PPIs in live cells using the MRC. A) Schematic illustrating the principle of visualizing PPIs in live cells using MRC. By utilizing the CRISPR‐dCas9 and SunTag system, sfGFP labeled target protein 1 was enriched to a designated genomic locus, to form bright spots, and mCherry labeled target protein 2 was recruited to the same sites to form colocalized spots due to its interaction with protein 1. B) Representative live‐cell imaging of MRC for SpyCatcher‐SpyTag and bJun‐bFos interactions. Corresponding constructs were co‐transfected into SunTag10x‐dCas9‐HEK293T cells (HEK293T cells stably expressing dCas9 with 10xGCN4), and images were obtained 24 h later. All images are maximum intensity projections from z‐stacks. SpyCatcher fused to scFv‐sfGFP is enriched at designated genomic loci, forming green spots. Fusion of mCherry‐labeled SpyTag with SpyCatcher results in stable covalent binding and colocalized red spots. bJun fused to sfGFP is enriched at designated genomic loci, forming green spots. Fusion of mCherry‐labeled bFos with bJun leads to non‐covalent interaction and colocalized red spots. As controls, bJun with SpyTag and SpyCatcher with bFos did not form colocalized spots. Dashed boxes show enlarged condensate images, and the right panels correspond to the fluorescence intensity line profiles of the enlarged images. All scale bars, 3 µm. C) The proportion of cells with successfully formed spots by recruiting SpyCatcher and bJun, n = 100 cells. D) The distribution percentage of the number of spots of SpyCatcher and bJun, n = 100 cells. E) Bar graphs show the mean ± standard deviation (SD) percentage of colocalized spots among the corresponding proteins, with three biological replicates and 15 cells per replicate. Sc: SpyCatcher; St: SpyTag; bJ: bJun; bF: bFos. Comparisons between two groups were performed using a t‐test. “ns” indicates no significance (*p* > 0.05). Since bJun does not interact with SpyTag and SpyCatcher does not interact with bFos, no colocalized spots were observed in these combinations. F) Box plots representing the ratio of SNR values for SpyCatcher, SpyTag, bJun, and bFos. Sc: SpyCatcher; St: SpyTag; bJ: bJun; bF: bFos. Sc/St indicates the SNR value of SpyCatcher spots divided by that of SpyTag spots. bJ/bF indicates the SNR value of bJun spots divided by that of bFos spots. n = 5 colocalized spots. “ns” indicates no significance (*p* > 0.05).

MRC utilizes CRISPR‐dCas9^[^ ^33^, ^34^, ^35^ ^]^ and SunTag^[^ ^36^ ^]^ to recruit bait proteins to the designated genomic loci, leading to localized protein clustering. As a result, proteins that interact with the bait were co‐recruited to the same loci, forming colocalized bright spots (**Figure** **1**A). We selected SpyCatcher, SpyTag, bJun, and bFos as subjects for our study.^[^ ^5^, ^37^ ^]^ We selected sgRNA *MUC4^‐272 kb^*, which targets a region ≈272 kb upstream of *MUC4* and contains ≈153 repeats. This sequence has been proven to label the corresponding genomic locus.^[^ ^38^ ^]^

To visualize the PPIs, we fused the target bait protein SpyCatcher to the C‐terminus of scFv‐sfGFP and SpyTag to the N‐terminus of mCherry. These fusion proteins were equipped with nuclear localization signals (NLS). We transfected the corresponding vector into SunTag10x‐dCas9‐HEK293T cells (HEK293T cells stably expressing dCas9 with 10xGCN4). Using dCas9 carrying 10xGCN4, guided by sgRNA *MUC4^‐272 kb^*, we enriched scFv‐sfGFP‐SpyCatcher at the corresponding genomic loci containing this designated genomic repetitive sequence, forming bright spots. As SpyTag‐mCherry can interact and bind with SpyCatcher, it was also recruited to the same genomic loci, resulting in the colocalization of two fluorescent wavelengths as merged bright spots (Figure 1A,B). Similarly, bFos and bJun formed colocalized dual spots (Figure 1B).

The efficiencies of SpyCatcher and bJun in generating bright spots at the genomic loci were relatively high and mainly formed two bright green spots (Figure 1C,D). Generally, the higher the binding capability between two proteins, the higher the proportion of colocalized spots in the cell. The dot signal‐to‐noise ratio (SNR) value represents the fluorescence intensity of the enriched protein at specific points. Theoretically, the higher the binding capability between interacting proteins, the more proteins are recruited and stabilized, leading to higher SNR values for the recruited protein. Therefore, the SNR ratio between the bait protein and the recruited interacting protein, averaged over multiple repeats, can be used to initially assess the binding capability between them. All data in the manuscript include the mean values and corresponding error margins (e.g., standard deviation) for SNR ratios and colocalization percentages, as presented in Tables S2 and S3 (Supporting Information), with additional information on the colocalization of interacting proteins provided in Table S1 (Supporting Information). As Figure 1E,F shown, the colocalization percentages and SNR ratios for SpyCatcher‐SpyTag and bJun‐bFos were not significantly different. We separately co‐ transfected scFv‐sfGFP‐bJun with SpyTag‐mCherry and scFv‐sfGFP‐SpyCatcher with bFos‐mCherry as controls, and the results indicated the absence of colocalized spots between them (Figure 1B,E). This indicates that proteins that do not interact do not form colocalization points. This indicates that the proportion of colocalized spots and the SNR ratio can serve as effective indicators for the preliminary estimation of binding affinities of PPIs.

We further validated the interactions between various proteins (Figure S1, Supporting Information), many of which are important drug targets.^[^ ^39^ ^]^ Notably, proteins such as P53, PICK, and BCL2 can self‐oligomerize, leading to the formation of numerous spots in the nucleus, which also colocalize with their interacting proteins. From these results, it is evident that this method has a broad applicability and a high positive detection rate for interacting proteins, making it suitable for various types of proteins.

### 2.2 Using MRC to Visualize and Validate PPIs within Multi‐Protein Complexes and to Compare Protein–Protein Binding Capability in Live Cells

**Figure 2:**
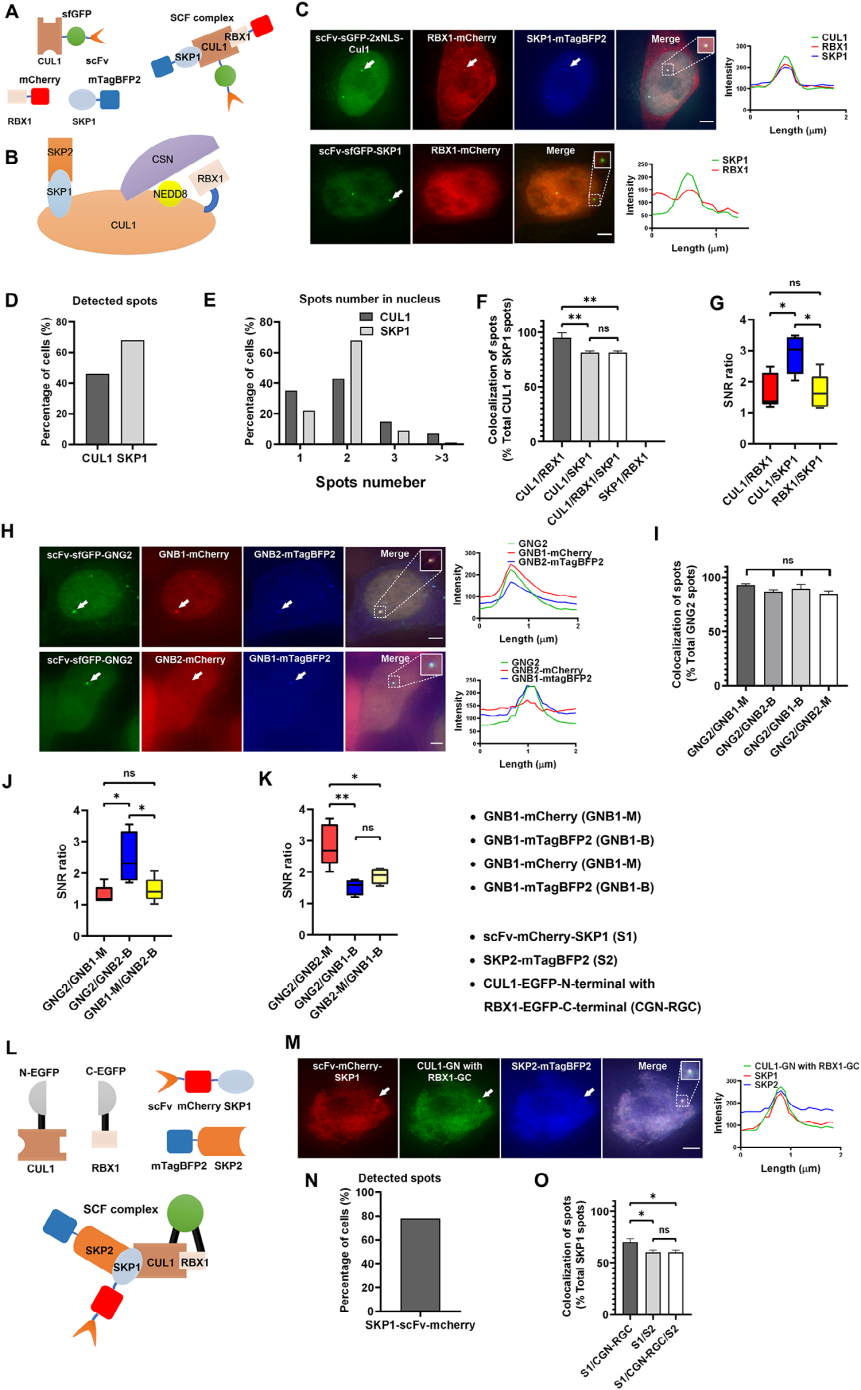
Visualizing the PPIs within multi‐protein complexes in live cells using MRC. A) Schematic of using MRC to visualize the interaction between CUL1, RBX1, and SKP1 within the SCF protein complex in live cells. CUL1 fused with scFv‐sfGFP is enriched at designated genomic loci, forming green spots. RBX1 and SKP1, both fused with mCherry and mTagBFP2, respectively, interact with CUL1 and are co‐recruited to corresponding loci, forming colocalized red and blue spots. B) Schematic diagram of the SCF complex. C–G) Application of MRC to confirm the interactions among multiple components of the SCF protein complex in living cells. C) The representative images show the visualization of protein interactions in the SFC complex using MRC. All corresponding constructs were co‐transfected into SunTag10x‐dCas9‐HEK293T cells, and all images were obtained 24 h later. Dashed squares indicate enlarged condensate images, and the right panel shows the corresponding fluorescence intensity profiles. All scale bars, 3 µm. D–G) corresponding to (C). D) The proportion of cells with formed spots by recruiting CUL1 and SKP1, n = 100 cells. E) The distribution percentage of the number of spots of CUL1 and SKP1, n = 100 cells. F) Percentage of colocalized spots among CUL1, RBX1, and SKP1. Graphs represent mean ± SD with three biological replicates, and each replicate contains 15 cells. Group comparisons were performed using Tukey's HSD test. ** indicates *p* < 0.01.“ns” indicates no significance (*p* > 0.05). SKP1 and RBX1 do not directly interact, thus, no co‐localized spots were formed. G) Box plot represents the ratio of SNR values among CUL1, RBX1, and SKP1. CUL1/RBX1: SNR values of CUL1 spots divided by those of RBX1 spots; CUL1/SKP1: SNR values of CUL1 spots divided by those of SKP1 spots; RBX1/SKP1: SNR values of RBX1 spots divided by those of SKP1 spots. *n* = 5 co‐localized spots. Group comparisons were performed using Tukey's HSD test * indicates *p* < 0.05. “ns” indicates no significance (*p* > 0.05). H–K) Application of MRC in the GPCR complex to compare relative binding affinities, using GNB1 and GNB2 competitively binding to GNG2 as an example. H) Representative images illustrating the utilization of the MRC for comparing the binding capacities of G protein β family members GNB1 and GNB2 with GNG2. Images were obtained 24 h after transfecting SunTag10x‐dCas9‐HEK293T cells. Dashed squares indicate enlarged condensate images. The corresponding fluorescence intensity profiles of the enlarged images are shown in the right panel. All scale bars, 3 µm. I–K) corresponding to (H). I) Percentage of colocalized spots among GNG2, GNB1, and GNB2. Graphs show the mean ± SD with three biological replicates, and each replicate contains 15 cells. Group comparisons were performed using Tukey's HSD test. “ns” indicates no significance (*p* > 0.05). No significant differences were observed between groups. J,K) Box plots represent the ratio of SNR values between GNG2, GNB1, and GNB2. (n = 5 colocalization points). GNG2/GNB1‐M: SNR value of GNG2 spot divided by that of GNB1‐M spot. Group comparisons were performed using Tukey's HSD test. * *p* < 0.05. ** *p* < 0.01. “ns” no significance, *p* > 0.05. L–O) Demonstration of MRC combined with BiFC for analyzing interactions among more than three proteins. L) Schematic of the principle of combining MRC with BiFC for visualization of interactions in multi‐protein complexes. SunTag10x‐dCas9‐HEK293T cells were transfected, and images were obtained after 24 h. SKP1 fused with scFv‐mCherry is enriched at designated genomic loci, forming red spots. CUL1 fused with EGFP at the N‐terminal (CUL1‐GN) and RBX1 fused with EGFP at the C‐terminal (SKP1‐GC) generate green fluorescence upon BiFC, and they are recruited by SKP1 to form co‐localized green spots. Additionally, SKP2 fused with mTagBFP2 is also recruited by SKP1, forming co‐localized blue spots. M) The representative images show the visualization of protein interactions in the SFC complex using MRC combined with BiFC. All corresponding constructs were co‐transfected into SunTag10x‐dCas9‐HEK293T cells, and images were obtained after 24 h. Dashed squares indicate enlarged condensate images, and the right panel shows the corresponding fluorescence intensity profiles. Scale bar, 3 µm. (N) and (O) corresponding to (M). N) The proportion of cells with successfully formed spots by recruiting SKP1‐scFv‐mCherry, n = 100 cells. O) Percentage of colocalized spots among the corresponding proteins. Graphs show the mean ± SD with three biological replicates, and each replicate contains 15 cells. Group comparisons were performed using Tukey's HSD test. * *p* < 0.05. “ns” no significance, *p* > 0.05.

In addition to visualizing the interactions between two proteins, we extended this system to investigate multiprotein interactions in complexes. SCF (SKP1‐Cullin‐F‐box protein) and SCF‐like complexes belong to the largest family of ubiquitin‐protein ligases.^[^ ^40^ ^]^ The crystal structure of the SCF complex revealed that CUL1 is an elongated protein with its N‐terminus binding to SKP1 and its C‐terminus binding to RBX1^[^ ^40^ ^]^ (**Figure** **2**B).

To study the SCF complex in live cells, we fused CUL1 with scFv‐sfGFP, RBX1 with mCherry, and SKP1 with mTagBFP2 and co‐transfected them with sgRNA *MUC4^‐272 kb^* into dCas9‐SunTag10x‐293T cells (Figure 2A). The results showed that scFv‐sfGFP‐CUL1 was recruited to the corresponding genomic loci, forming bright spots (Figure S2A, Supporting Information). However, the larger molecular weight of CUL1, resulting in lower nuclear entry efficiency (Figure S2A,E, Supporting Information). To optimize the nuclear entry efficiency of larger proteins, we added an NLS. The results showed that 2xNLS enhanced the nuclear entry efficiency (Figure 2C; Figure S2A,E, Supporting Information), resulting in higher positive rates of spot appearance and colocalization with RBX1 (Figure 2D; Figure S2B,D, Supporting Information). CUL1 and SKP1 are primarily recruited to the genome, forming two main spots (Figure 2E).

To further optimize the labeling efficiency while minimizing non‐specific signals, we systematically compared different GCN4 repeat numbers. We compared 2xGCN4, 10xGCN4, and 24xGCN4 (2x, 10x, and 24x). The 24x repeat amplification showed higher positive rates of nuclear spot appearance (Figure S3A,B, Supporting Information) and a higher colocalization ratio compared to the 2x magnification (Figure S3D, Supporting Information). There was no significant difference in the SNR ratios among the three groups (Figure S3E, Supporting Information). Nonetheless, 24x resulted in more than three spots (Figure S3C, Supporting Information), indicating that the number of non‐targeted chromosomal spots was also higher. Taking all these factors into consideration, we used 10xGCN4 for subsequent experiments.

To further enhance signal intensity at the genomic locus, we increased the number of recruitment sites by extending the repeat region. In the original region, we further designed a sgRNA *MUC4^‐272kb‐42^* with 42 repeats near *MUC4^‐272 kb^* locus and co‐transfected it along with *MUC4^‐272 kb^* into cells. An increased number of repeats recruits additional target proteins. The results showed that the number of positive spots increased compared with the original condition, with no significant difference in the colocalization ratio (Figure S4A,B,D, Supporting Information). But the addition of 42 repeats significantly increased the SNR (Figure S4E, Supporting Information). To help other researchers quickly adapt the MRC system to their specific target proteins, we have provided a troubleshooting guide in Table S6 (Supporting Information), which systematically summarizes potential issues encountered with the system and corresponding optimization strategies.

As shown in Figure 2C, RBX1‐mCherry and SKP1‐mTagBFP2 were recruited by CUL1 to form colocalized spots. The colocalization ratio of CUL1 and RBX1 was greater than that of SKP1 (Figure 2F). The SNR ratio between CUL1 and RBX1 spots was lower than the SNR ratio between CUL1 and SKP1 spots (Figure 2G), indicating that CUL1 recruited more RBX1, forming a more stable colocalization.

Interestingly, when co‐transfected with scFv‐sfGFP‐SKP1 and RBX1‐mCherry, RBX1 did not form bright spots when recruited by SKP1 (Figure 2C,F). This indicates that the two proteins do not directly interact but instead form a complex mediated by CUL1. We speculated that free CUL1 expressed in the nucleus may not be sufficient to recruit adequate amounts of RBX1 and SKP1 to form bright spots. When TetON‐SKP1‐mCherry was not expressed, CUL1 did not colocalize with SKP2. However, upon addition of Docx to induce SKP1 expression, CUL1 colocalized with both SKP1 and SKP2 (Figure S5, Supporting Information). Thus, we inferred that CUL1 interacts directly with SKP1, whereas SKP1 interacts with SKP2. Similarly, we took the interaction between CUL1 and the COP9 Signalosome (CSN) complex^[^ ^41^ ^]^ as an example. We observed that CUL1 colocalized with CSN2 in the complex but not with CSN5 (Figure S6A,B, Supporting Information). This indicates that CUL1 directly interacts with CSN2 but not with CSN5.

Using the MRC method, we made an interesting discovery that upon overexpression of RBX1, NEDD8 exhibited higher colocalization with CUL1, indicating that RBX1 enhanced the binding of NEDD8 to CUL1 (Figure S7A–C, Supporting Information). This suggests that statistical analysis can be employed to assess the real‐time contribution of individual proteins to complex stability within multi‐protein complexes.

We used G‐protein‐coupled receptors (GPCRs) as an example to compare the interaction‐binding capabilities of different G‐protein complex members. Some GPCRs couple with a single G protein, while many can interact with more than one G protein, typically exhibiting different coupling efficiencies depending on the G protein subtype.^[^ ^42^, ^43^ ^]^ As depicted in Figure 2H, GNB1 and GNB2 can bind to GNG2 simultaneously. From Figure 2I–K, it is evident that, regardless of the type of fluorescent protein fusion, GNB1 exhibited a higher colocalization percentage with GNG2 and a higher SNR value compared to GNB2. This indicates that GNB1 has a stronger binding capability for GNG2 than for GNB2. GNB3T is the loss of 41 amino acids and one WD repeat domain from the Gβ subunit.^[^ ^44^ ^]^ Compared to GNB1 and GNB2, GNB3T does not exhibit colocalization with GNG2 (Figure S8G,J, Supporting Information). GNG2 stably interacted with GNB1 and simultaneously formed a ternary complex with GNAI1 (Figure S8A–E, Supporting Information). The proportion of cells with colocalization of GNG2 and GNAQ was relatively low compared to that of GNG2 and GNAI1, and the SNR ratio was lower for GNAQ (Figure S8F,H,I, Supporting Information). This shows that the binding capacity of the GNG2‐GNB1 complex to GNAI1 is greater than that to GNAQ.

In the future, additional spectrally distinct fluorescent proteins can be incorporated to enable simultaneous visualization of more PPIs. However, as the commonly used imaging setup in our laboratory typically involves three channels, we further developed a method to enable simultaneous visualization of multiple protein interactions under three channels configuration. BiFC allows the visualization of the interaction between two proteins in a single fluorescence channel. To visualize additional protein complexes, we combined BiFC and MRC. Using this approach, we simultaneously observed additional protein complexes (Figure 2L). Specifically, we recruited scFv‐mCherry‐SKP1, and the direct interaction between CUL1 and RBX1 resulted in green fluorescence. CUL1 was recruited by SKP1, forming colocalized green spots, whereas the interaction between SKP2‐mTagBFP2 and SKP1 resulted in colocalized blue spots (Figure 2M). Thus, the three‐color system allows simultaneous visualization of four protein complexes, and theoretically, the three‐channel setup can visualize up to six protein complexes. We also calculated the percentage of scFv‐mCherry‐SKP1 spots in the cell nucleus, spot distribution, and colocalization percentage ratios (Figure 2N,O).

### 2.3 Utilizing MRC to Induce the Formation of Biomolecular Condensates in Living Cells and Assessing the Contribution of Multi‐Protein Interactions to Promote Phase Separation

**Figure 3:**
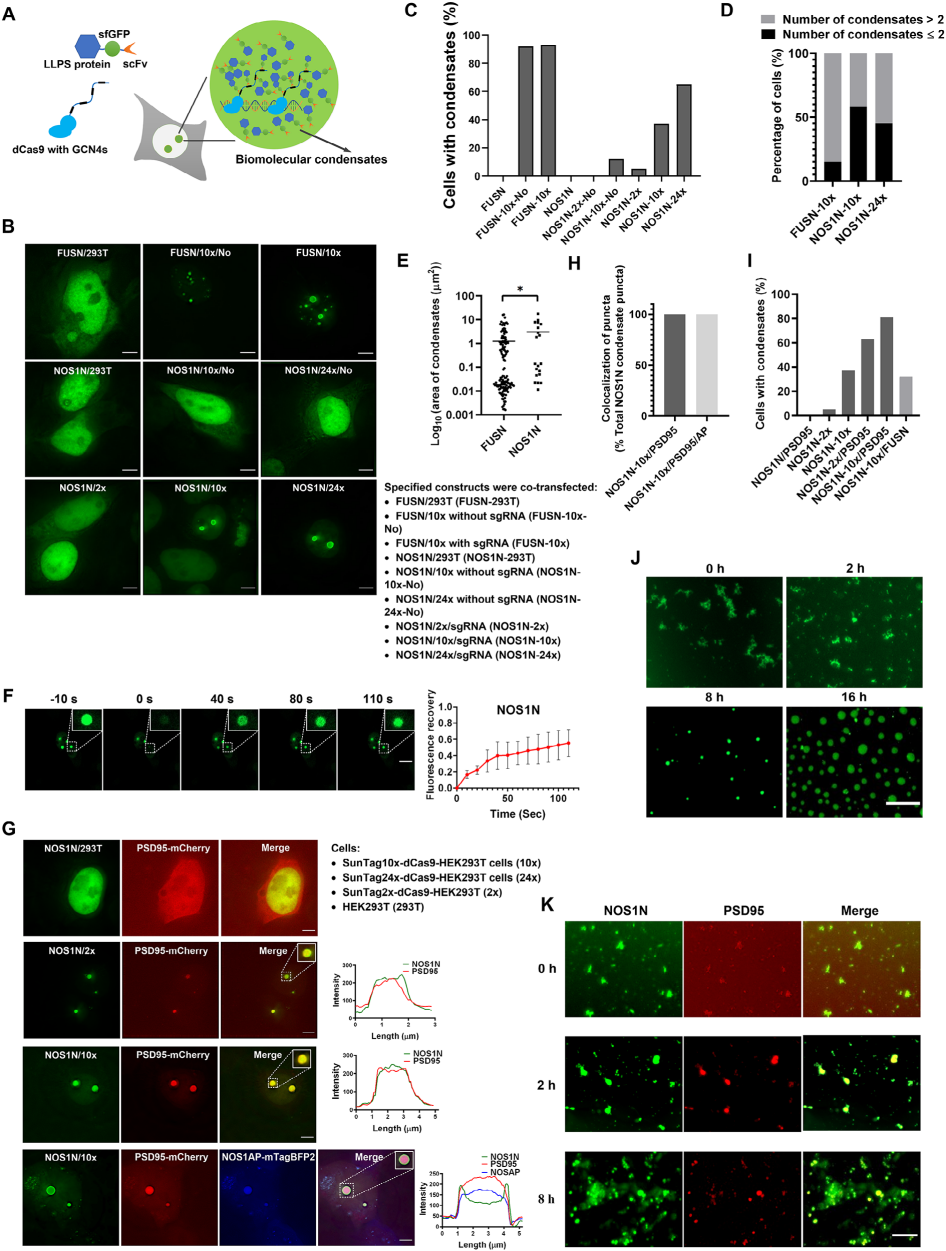
Inducing phase separation and assessing the contribution of multi‐protein interactions to promote phase separation using MRC in live cells. A) Schematic depicting the induction of phase separation in live cells using MRC. Phase‐separating proteins are recruited to designated genomic repeat sequences and drive phase‐separated biomolecular condensates formation through CRISPR‐dCas9 and SunTag. B–F) MRC induces phase‐separating proteins to form condensates in live cells. B) Representative images showing that the specified constructs were transfected into respective cells, and images were obtained after 24 h. All scale bars, 5 µm. 2x: SunTag2x‐dCas9‐HEK293T cells (HEK293T cells stably expressing dCas9 with 2xGCN4); 10x: SunTag10x‐dCas9‐HEK293T cells; 24x: SunTag24x‐dCas9‐HEK293T cells (HEK293T cells stably expressing dCas9 with 24xGCN4). C–E) corresponding to (B). C) Percentage of cells forming respective condensates (n = 100 cells). D) The distribution percentage of the number of respective condensates (n = 100 cells). E) Quantification of normalized condensate areas for FUSN and NOS1N (n = 5 cells), scatter plot represents log10 normalized values of condensate areas, each point represents one condensate. Comparison between the two groups was performed using a t‐test. * *p* < 0.05. F) FRAP analysis of NOS1N in cells. Dotted square shows the photobleaching region. The right panel represents quantification of fluorescence intensity changes over time after photobleaching of NOS1N condensates. Values represent mean ± SD of background‐subtracted fluorescence measurements (n = 5 condensates). Scale bar, 5 µm. G–I) MRC reveals that the NOS1 interactor PSD95 promotes the formation of NOS1N condensates. G) Representative images of the corresponding constructs transfected into SunTag10x‐dCas9‐HEK293T cells. Dotted square shows enlarged condensates images, and the right panel corresponds to the fluorescence intensity line plot of the enlarged region. All scale bars, 3 µm. (H) and (I) corresponding to (G). H) Percentage of co‐localized of NOS1N with PSD95 and NOS1AP in the presence of NOSN condensates formation. Graphs show the mean ± SD with three biological replicates, and each replicate contains 15 cells. AP: NOS1AP. I) Percentage of cells with condensates formed after co‐transfected NOS1N and PSD95 with the respective constructs (n = 100 cells). J,K) In vitro validation of NOS1N condensate formation. J) Representative images of in vitro condensates formation and time‐lapse microscopy of condensates. Scale bar, 100 µm. K) Time scale images representation of condensates formation by NOS1N protein mixed with PSD95 protein in vitro. Scale bars, 100 µm.

The molecular basis of various proteins and nucleic acids undergoing phase separation in cells involves multivalent low‐affinity interactions.^[^ ^45^ ^]^ Using MRC, we induced phase‐separating proteins to form locally high concentrations near the target genomic loci, leading to the formation of phase boundaries exceeding the saturation concentration, nucleation, and initiation of condensate growth (**Figure** **3**A). While studying the NOS1‐PSD95 complex using MRC, we discovered that the N‐terminal 235 amino acids of NOS1 (NOS1N) underwent phase separation (Figure 3B,C). The phase separation of NOS1N was further validated using fluorescence recovery after photobleaching (FRAP) and in vitro experiments (Figure 3F,J). In the absence of SunTag, no condensates were formed when only FUSN (1‐215 aa) and NOS1N were individually transfected (Figure 3B,C). Without sgRNA, scFv‐FUSN is not recruited to genomic loci, but can broadly induce phase separation when recruited by the 10xSunTag system. In contrast, only a small fraction of cells underwent phase separation when NOS1N was transfected (Figure 3B,C). This finding suggests that patients with FUSN are more prone to developing phase separation. After introducing sgRNA, the proportion of cells exhibiting NOS1N condensates significantly increased, and the 24x SunTag produced more condensate than the 10x SunTag (Figure 3B–D). When comparing FUSN with NOS1N, FUSN formed more condensates but with a smaller average area (Figure 3E). Protein size can influence condensate properties, as evidenced by FUS320 (1‐320 aa) forming larger condensates than FUS460 (1‐460 aa) (Figure S9A,B, Supporting Information). Additionally, because of the generally higher protein expression levels in HEK293T cells than in HeLa cells (Figure S9C,D, Supporting Information), increased protein expression favors condensates formation.

MRC can be used to study protein complexes and the roles of interacting proteins within these complexes during phase separation. Using MRC, we demonstrated that PSD95's PDZ2 domain interacts with both NOS1 PDZ and NOS1AP (Figure 3G,H; Figures S10C and S11A, Supporting Information), which is consistent with previous findings.^[^ ^46^, ^47^, ^48^ ^]^ PDZ domains are modular protein–protein interaction domains specifically designed to bind to the C‐terminal peptide motifs of other proteins and are commonly involved in assembling large molecular complexes.^[^ ^49^ ^]^ The introduction of PSD95, which introduced new PDZ interactions, enhanced the proportion of cells exhibiting condensation (Figure 3H,I). In contrast, co‐expression of NOS1N with the non‐interacting protein FUSN does not increase the probability of NOS1N condensate formation (Figure 3I). In vitro experiments also showed that adding PSD95 can accelerate the formation of NOS1N condensates (Figure 3K). In addition, ZL006, an inhibitor of the interaction between NOS1 and PSD95, was used to disrupt the interaction between NOS1N and PSD95.^[^ ^50^ ^]^ Upon disruption of this interaction, a marked reduction in condensate formation was observed. This result serves as a reverse functional validation, supporting the critical role of the NOS1N–PSD95 interaction in promoting condensate formation (Figure S12, Supporting Information).

Previous studies have indicated that proteins, such as GRB2, can undergo rapid phase separation in the presence of SOS1.^[^ ^51^ ^]^ Similarly, using the MRC method, we demonstrated that SOS1 promoted GRB2 condensates formation. In the absence of SOS1, GRB2 forms punctate spots (Figure S13A–C, Supporting Information). We preliminarily assessed the direct binding affinities of the NOS1N/PSD95 and GRB2/SOS1 pairs by comparing the SNR values of their condensate‐like puncta. The results showed that the average SNR ratio for NOS1N/PSD95 puncta was 1.42 ± 0.19, and for GRB2/SOS1 it was 1.46 ± 0.19, indicating strong interaction affinities for both pairs (Figure S13D and Table S3, Supporting Information).

We further evaluated in live cells how different expression levels (i.e., varying endogenous stoichiometries) influence LLPS behavior. Specifically, we categorized cells co‐expressing NOS1N and PSD95 into two groups: those exhibiting typical condensates (i.e., large, round bright foci) and those displaying only small puncta. For each group, we measured the nuclear fluorescence signal intensity (SNR value) of the two proteins as a proxy for their relative expression levels. The results showed that nuclear PSD95 expression was significantly higher in cells that formed condensates compared to those that did not (Figure S15A,B, Supporting Information). In contrast, the nuclear expression level of NOS1N did not differ significantly between the two groups (Figure S15C, Supporting Information).

Next, we analyzed the phase separation capability of the PDZ domains of NOS1N and PSD95 using MRC analysis. According to the predictions from the PONDR software, within these 235 amino acids, besides the PDZ domain, there was also a segment of Intrinsically Disordered Region (IDR) (Figure S10B, Supporting Information). We separately validated whether the PDZ region and IDR could form phase‐separated condensates using MRC. The results showed that NOS1 PDZ could self‐oligomerize and form irregular aggregates, but not perfectly round condensates (Figure S10A, Supporting Information). FRAP experiments indicated partial recovery but lacked rapid recovery capacity (Figure S10D, Supporting Information). In contrast, IDR exhibited an even more irregular morphology (Figure S10A, Supporting Information). This indicates that neither of the two regions individually formed condensates. However, the simultaneous presence of both regions can induce phase‐separated condensate formation.

PSD95 and PSD95 PDZ2 domains are also similar cases as NOS1 PDZ (Figure S11A,B, Supporting Information). We hypothesized that the PSD95 and PSD95 PDZ2 domains may be involved in aggregation or gel formation rather than the formation of liquid condensates.

In the future, the MRC system could be extended to the cytoplasm. For example, dCas13 could be used to target RNAs containing repetitive sequences, enabling the recruitment of proteins in the cytoplasm to form detectable spots or induce condensate formation.

### 2.4 Rapid Assessment of Protein Domains with Condensate Forming Potential Using MRC

**Figure 4:**
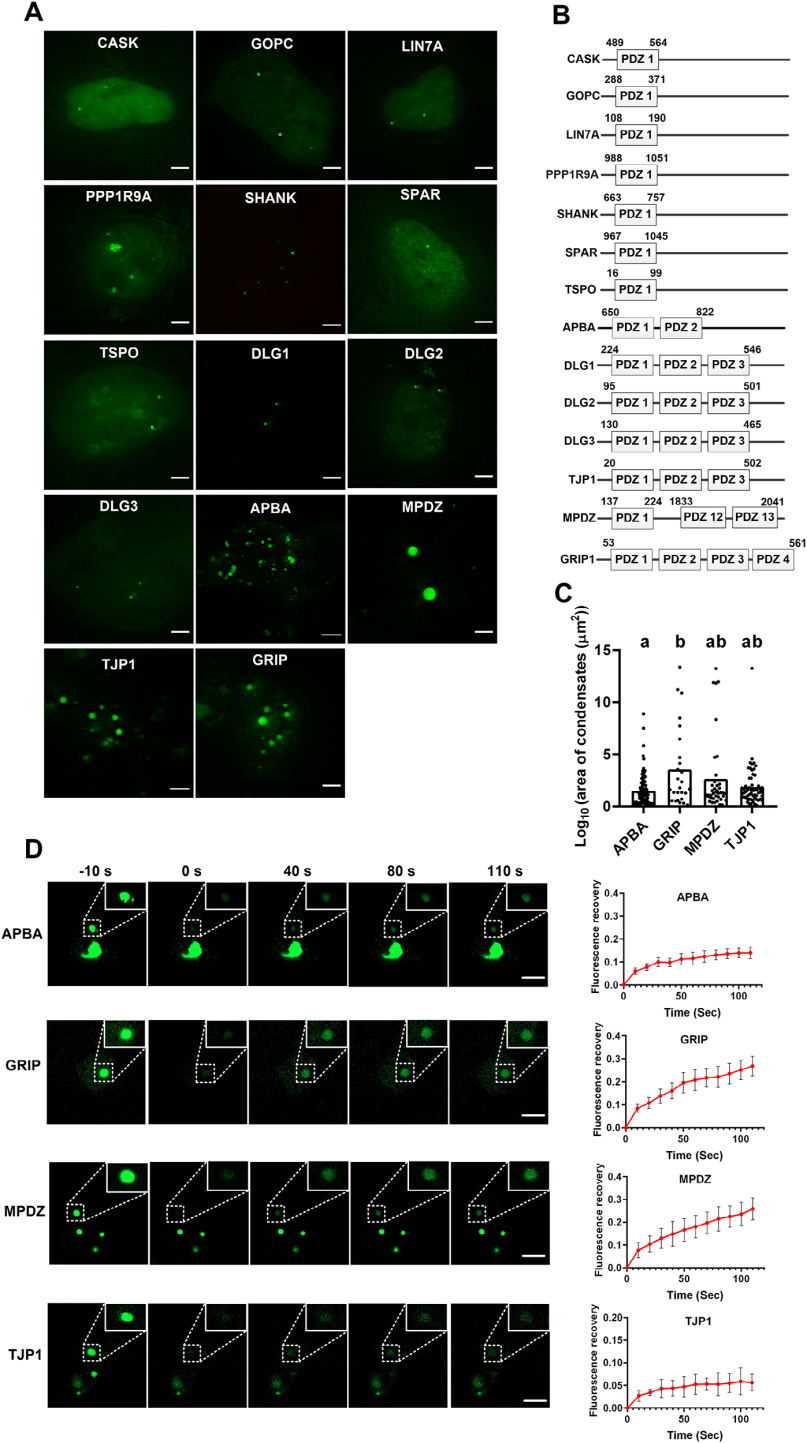
Screening of PDZ domains capable of phase separation using MRC. A) Representative images of various PDZ domains fused with scFv‐sfGFP under the SunTag24x system. The cells were transfected with the respective PDZ domain constructs in SunTag24x‐dCas9‐HEK293T cells, and images were obtained after 24 h. All scale bars, 3 µm. B) Schematic representation of the amino acid positions of the PDZ domains in the genes used for validation. C) Scatter plot showing the distribution of spot areas (log10 normalized values) for APBA, GRIP, MPDZ, and TJP1. Each dot represents one condensate, n = 5 cells. Distributions with the same letter are not significantly different according to Tukey's HSD test (*p* < 0.05). Each dot represents a single condensate; n = 5 cells. D) FRAP analysis of APBA, GRIP, MPDZ, and TJP1 in cells. Dotted square indicates the photobleached region. All scale bars, 5 µm. The right graph represents the quantification of fluorescence measurements of respective condensates over time after photobleaching. Values represent the mean ± SD after subtracting background fluorescence (n = 5 cells).

The PDZ domains exhibit multivalency^[^ ^49^ ^]^ and are potential regions for phase separation. We selected the PDZ domains from 14 proteins, all of which are important drug targets.^[^ ^49^ ^]^ Among them, eight proteins contained a single PDZ domain, whereas seven proteins contained multiple PDZ domains. We determined the occurrence of phase separation by assessing whether the condensates were circular in shape and size and by conducting FRAP experiments.^[^ ^27^ ^]^ **Figure** **4**B shows the fused PDZ segments, where MPDZ contains multiple PDZ domains, but only PDZ1, PDZ12, and PDZ13 were fused in our study. The results revealed that APBA, GRIP, MPDZ, and TJP1 formed relatively well‐defined and large circular condensates (Figure 4A,C). Based on the FRAP results, APBA, GRIP, and MPDZ exhibited varying degrees of recovery, with TJP1 exhibiting the slowest recovery (Figure 4D). Preliminary evidence suggests that the PDZ domains of APBA, GRIP, MPDZ, and PDZ1 exhibit features consistent with liquid‐like behavior. TJP1, on the other hand, forms spherical structures but lacked liquid recovery after photobleaching, indicating that TJP1 condensates exhibited more solid‐like gel characteristics. We analyzed the circularity of condensates formed by the candidate proteins (circularity = 4π × area / perimeter^2^, ranging from 0 to 1). The results showed that NOS1N condensates generally exhibited high circularity values, approaching 1, which is close to an ideal spherical shape (Figure S14, Supporting Information), supporting their identity as typical condensate‐like structures. We also extracted representative quantitative FRAP parameters for each protein, including Mobile Fraction (indicating the proportion of dynamic molecular exchange within the condensates); %Recovery (reflecting the actual extent of fluorescence recovery during the experiment). The results showed that NOS1N exhibited the highest mobility (mobile fraction: 0.551 ± 0.180; % recovery: 55.11 ± 18.02%), while TJP1 showed the lowest (0.056 ± 0.020; 5.58 ± 1.97%). GRIP and MPDZ displayed intermediate mobility levels, indicating significant differences in the dynamic properties among these six proteins (Table S7, Supporting Information).

It should be noted that although the MRC system allows for rapid observation of condensate formation by proteins with potential phase separation capability in live cells and enables convenient FRAP analysis, this alone is not sufficient to conclusively confirm phase separation. Further validation with additional assays, such as in vitro condensate formation experiments, is necessary, and results should be interpreted with caution. MRC is better suited as an efficient preliminary screening tool, facilitating the rapid identification of candidate proteins with potential phase separation capability without the need for labor‐intensive in vitro assays for every target.

### 2.5 Programming Spatial Separation Sites for Two Phase‐Separating Proteins and Studying the Interactions of Condensates

We further extended the MRC method by introducing MoonTag and dSaCas9, which enabled the recruitment of phase‐separating proteins to designated genomic loci and the generation of phase‐separated condensates. MoonTag is an orthogonal genetically encoded antibody‐epitope fluorescent labeling strategy in addition to SunTag, which consists of a 15‐amino acid gp41 peptide and its affinity binding with a 123‐amino acid gp41 antibody.^[^ ^52^ ^]^ The SaCas9 is a smaller homolog of Cas9 from Staphylococcus aureus that recognizes a 5′‐NNGRRT‐3′ PAM (R representing A or G), and dSaCas9 is a nuclease‐deficient variant of SaCas9.^[^ ^38^, ^53^ ^]^

**Figure 5:**
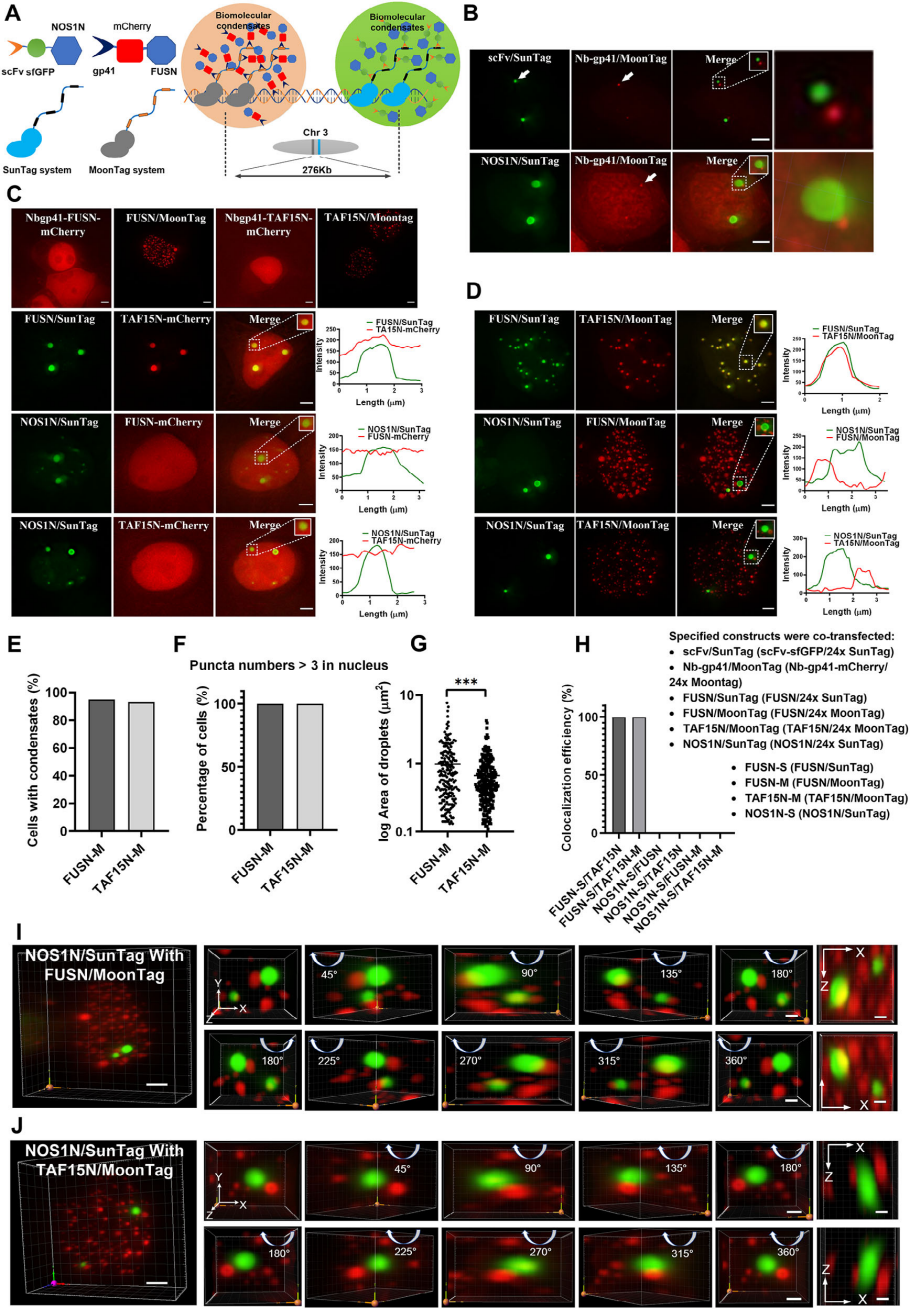
Simultaneous programming of two phase‐separating protein condensates at designated spatial loci using MRC. A) Schematic representation of inducing condensates formation of two phase‐separating proteins at adjacent loci using the MRC method. The dCas9‐SunTag24x system forms condensates at a designated locus, while the dSaCas9‐MoonTag24x system forms another condensates at the nearby locus. B–H) The MRC approach utilizes the SunTag and MoonTag systems to simultaneously program two phase‐separating protein condensates at designated spatial loci in cells. Quantifications include parameters related to the formation of the second type of condensates upon introduction of the MoonTag system. B–D) All respective constructs were co‐transfected into HEK293T cells, and images were obtained after 24 h. Dashed boxes show enlarged condensate images, and the right panels correspond to the fluorescence intensity line profiles of the enlarged images. B) Representative images of condensates formed by the dCas9‐SunTag24x system and the dSaCas9‐MoonTag24x system labeled spots nearby. All scale bars, 3 µm. C) Representative images of condensates formed by MoonTag system. All scale bars, 3 µm. D) Representative images showing condensates of different proteins induced by the dCas9‐SunTag24x system and the dSaCas9‐MoonTag24x system at adjacent loci. All scale bars, 3 µm. E–G) corresponding to (C). E) Percentage of cells with condensates of FUSN/MoonTag24x and TAF15N/MoonTag24x (n = 100 cells). F) Percentage distribution of condensates of FUSN/MoonTag24x and TAF15N/MoonTag24x in each cell. G) Normalized quantification of condensate areas formed by FUSN and NOS1N in the MoonTag system (n = 5 cells). Scatter plot represents log10 normalized values of condensate areas. Each data point represents a condensate. Comparison between the two groups was performed using a t‐test. *** *p* < 0.001. H) corresponding to c‐d. Average percentage of co‐localized condensate signals formed by respective constructs. Three biological replicates were performed, with 15 cells per replicate. I,J) Representative images showing 3D visualization of I) NOS1N/SunTag condensates with FUSN/MoonTag condensates and J) NOS1N/SunTag condensates with TAF15N/MoonTag condensates. The right panel shows the corresponding 360‐degree rotating decomposed images. The NOS1N condensates do not merge with FUSN or TAF15N condensates. All scale bars, 1 µm.

Using the sgRNA *MUC4E*,^[^ ^38^ ^]^ we recruited MoonTag and phase‐separating proteins to the *MUC4* locus, which is located ≈272 kb from the SunTag recruitment site (*MUC4^‐272 kb^*) (**Figure** **5**A). Using this approach, we were able to simultaneously induce the phase separation of two different proteins in a modularly designed and programmable spatially directed manner. By generating phase separation at specific locations, we investigated the physical interactions between the different types of condensates. From Figure 5B, we observed that the NOS1N condensates did not engulf or wrap around Nb‐gp41 dots. The coexistence of intraphase proteins and aggregating Nb‐gp41 proteins appeared to be incompatible, as condensate growth was inhibited upon encountering heterotypic proteins.

We successfully induced phase separation and co‐condensates of FUSN and TAF15N (1‐208 aa) using the dSaCas9‐MoonTag24x system (Figure 5C,E). Multiple condensates were observed within a single cell (Figure 5F,G). As shown in Figure 5C,H, TAF15N‐mCherry was observed within the FUSN condensate, indicating a direct or indirect interaction between FUSN and TAF15N. Thus, after inducing the formation of two sets of condensates using FUSN/SunTag at the *MUC4^‐272 kb^* locus and TAF15N/ MoonTag at the *MUC4E* locus, FUSN and TAF15N did not form separate condensates (Figure 5D,H).

We further investigated whether the NOS1N/SunTag condensates interact with FUSN/MoonTag and TAF15N/MoonTag condensates. Figure 5D,H–J revealed that the NOS1N condensates formed near the *MUC4^‐272 kb^* locus did not merge with the FUSN or TAF15N condensates formed at the *MUC4E* locus. Although they grow into large condensates and are in close proximity, they are not in physical contact with each other. Based on this observation, we deduced that condensates formed by NOS1N do not interact with FUSN and that TAF15 condensates remain physically separated and maintain distinct, independent condensates in the cell. Furthermore, it can be inferred that the two condensates do not grow toward each other in their respective directions or make contact.

### 2.6 Programming Engineered Condensates in Living Cells Using MRC and Coupled with Optogenetic Tools to Make Target Proteins Enter the Condensates

**Figure 6:**
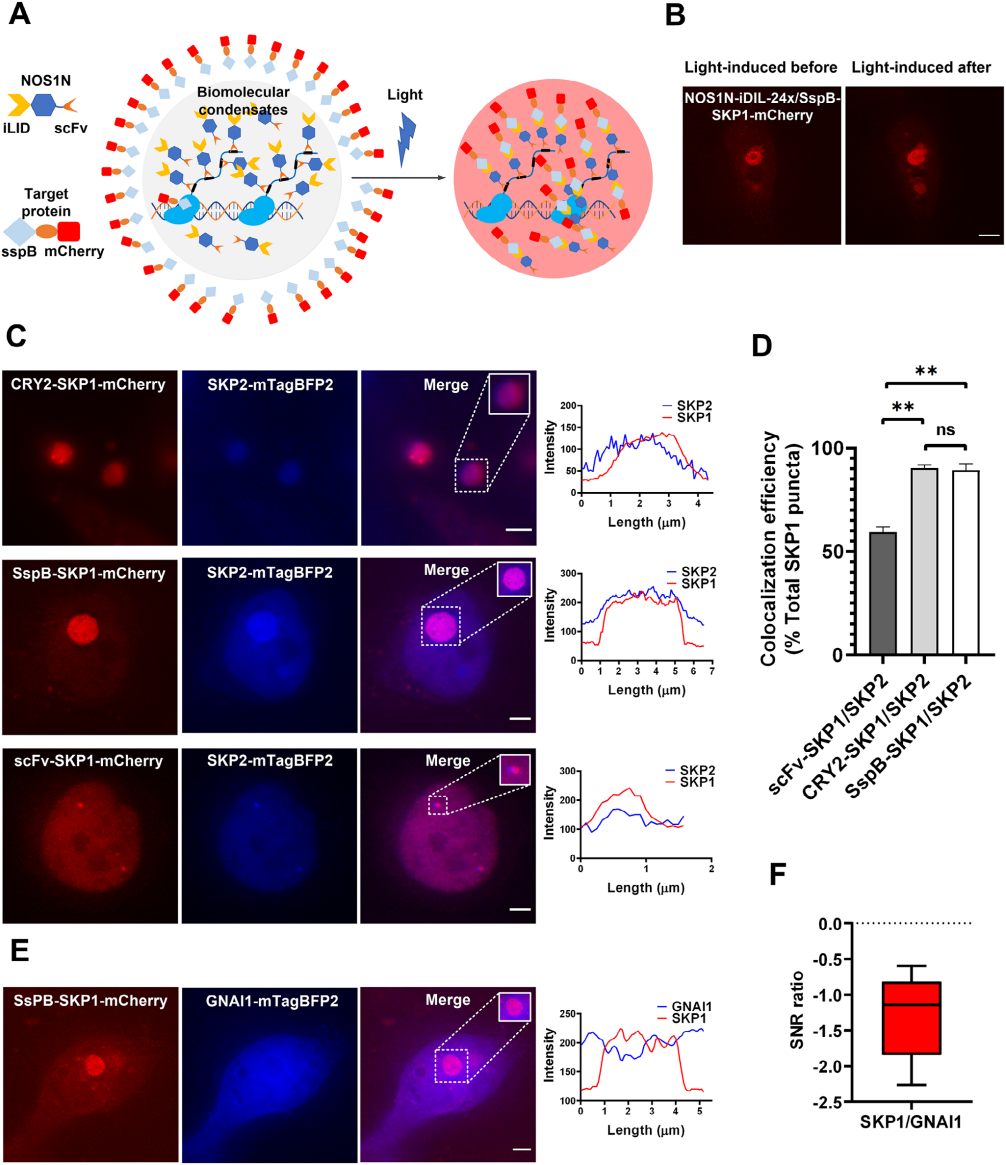
MRC combined with optogenetic tools enables programmable engineering of phase‐separated condensates that recruit target proteins. A) Schematic illustration of MRC combined with optogenetic elements to induce phase separation for non‐phase separating proteins. The phase‐separating protein (NOS1N) fused with iLID forms condensates. Upon exposure to blue light, iLID dimerizes with sspB, recruiting the target protein fused with sspB into the condensates. This process imparts the physical properties of the condensates to the target protein. B) Representative images showing the localization of ssPB‐mCherry in NOS1N condensates before and after illumination. Prior to illumination, some background ssPB‐mCherry interacts with NOS1N‐iLID, and after illumination, a large amount of ssPB‐mCherry enters NOS1N condensates. Scale bar, 3 µm. C) Representative images of indicated constructs transfected and all constructs were co‐transfected into SunTag10x‐dCas9‐HEK293T cells. Dashed boxes show enlarged condensate images, and the right panels correspond to the fluorescence intensity line profiles of the enlarged images. All scale bars, 3 µm. D) The colocalization percentage of scFv‐SKP1, CRY2‐SKP1‐scFv, and ssPB‐SKP1‐scFv with SKP2‐TagBFP2. Three biological replicates were performed, with 15 cells per replicate. Group comparisons were performed using Tukey's HSD test. *** *p* < 0.001. “ns” no significance, *p* > 0.05. E) Representative images demonstrate GNAi being isolated by SKP1 condensates. Scale bar, 3 µm. F) The ratio of SNR values between ssPB‐SKP1‐mCherry and GNAi‐mTagBFP2 condensate signals, n = 5 condensate signals. SKP1: ssPB‐SKP1‐mCherry, GNAi: GNAi‐mTagBFP2.

We envision first using the MRC system to form different types of condensates and then employing optogenetic tools to recruit exogenous target proteins into them, allowing the target proteins to utilize the properties of the pre‐formed condensates. We replaced sfGFP in scFv‐NOS1N with the optogenetic dimerization proteins iLID or CRY2.^[^ ^54^, ^55^ ^]^ Additionally, we fused ssPB or CIBN to the non‐phase separating protein SKP1. Both constructs, scFv‐NOS1N‐iLID and SKP1‐ssPB, were co‐transfected with sgRNA *MUC4^‐272 kb^* into SunTag10x‐dCas9‐HEK293T cells. Initially, scFv‐NOS1N‐iLID formed condensates at the targeted genomic *MUC4^‐272 kb^* site. Upon blue light activation, iLID and ssPB bound together, recruiting SKP1‐ssPB to scFv‐NOS1N‐iLID condensates (**Figure** **6**A–C). However, in both CRY2 and iLID optogenetic systems, there is pre‐existing background aggregation before illumination (Figure 6B). This could be attributed to the amplification of this background by dense condensates In the future, light conditions should be carefully controlled to avoid nonspecific activation. Low‐background mutants such as CRY2 (L348F) can significantly reduce dark‐state binding while retaining light responsiveness.^[^ ^56^ ^]^ Furthermore, optimized systems like CRY2clust can promote rapid and efficient homotypic oligomerization, thereby enhancing specificity and reducing background interference.^[^ ^57^ ^]^

This approach allowed us to introduce various proteins into the condensates, creating an intracellular environment for them to form membraneless condensates with artificial phase separation characteristics. In the NOS1N‐SKP1 condensates region, the fluorescence intensity of the GNAI1‐mTagBFP2 channel was lower than the surrounding fluorescence intensity, resulting in a negative SNR region that formed a circular “dark hole” reminiscent of a nucleolus (Figure 6E,F). Moreover, this method enables the modular design of different condensate types, allowing the synthesis of diverse condensates within cells, such as scFv‐NOS1N‐iLID and scFv‐FUSN‐iLID condensates (Figure S16, Supporting Information) to acquire the properties of these condensates.

We utilized optogenetic proteins to recruit target proteins into the condensates, resulting in larger and brighter signals than the point‐like recruitment achieved using the MRC method described in the previous section. Taking the SKP1‐SKP2 interacting protein as an example, the colocalization percentage of SKP2 with SKP1 within the condensates was higher (Figure 6C,D). Therefore, phase separation‐integrated MRC can be extended to study the interactions among proteins with weaker binding affinities.

### 2.7 Using Quantum Dots as Probes in Combination with MRC to Investigate the Physical Properties of Phase‐Separated Condensates in Living Cells

**Figure 7:**
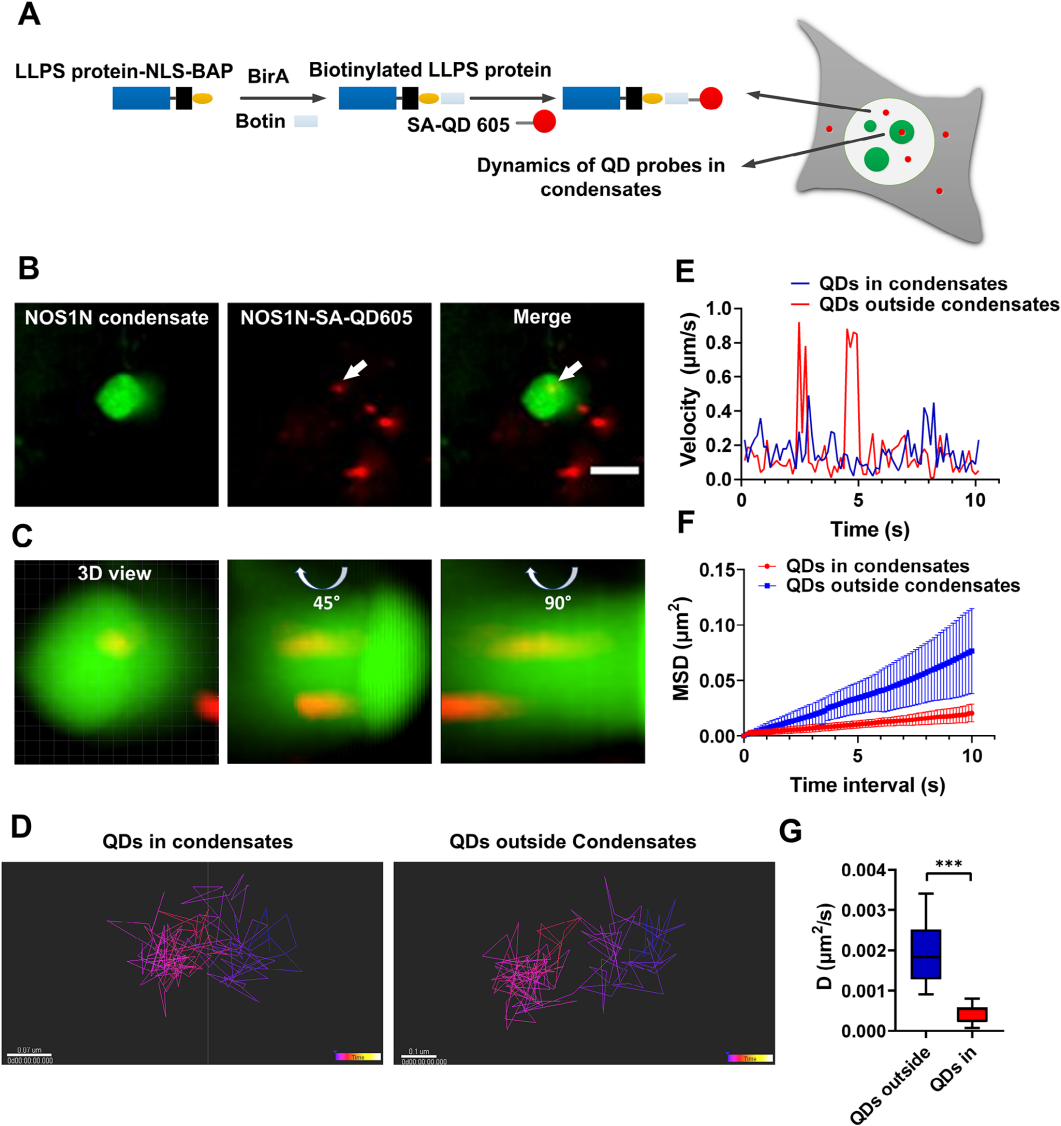
MRC coupled with QDs as probes for investigating the physical properties of phase‐separated condensates in live cells. A) Schematic representation of QDs as probes entering the nucleus to detect condensates. Streptavidin‐coated QDs bind to biotinylated phase‐separating proteins, are guided into the nucleus by NLS, and are then directed into condensates by the phase‐separating proteins. B) Representative images showing NOS1N‐NLS‐BAP‐QDs entering the nucleus are captured by NOS1N condensates. All corresponding constructs were co‐transfected into SunTag10x‐dCas9‐HEK293T cells. Scale bar, 3 µm. C) Local 3D imaging obtained from Z‐stack in b, indicates QDs entering the interior of the condensates. D) Trajectories of QDs inside and outside the condensates in b. The scale bar in the left image is 0.7 µm, while the scale bar in the right image is 1 µm. E) Corresponding to (B), time‐dependent displacement velocities of QDs inside and outside the condensates. F) Average MSD curves of QDs inside and outside the condensates, n = 15 trajectories. G) Boxplots of effective diffusion coefficients of QDs inside and outside the condensates. Comparison between the two groups was performed using a t‐test. *** *p* < 0.001.

Quantum dots (QDs) have unique optical properties, such as excellent brightness and remarkable photostability. Single QDs are sufficiently bright for long‐term single‐particle tracking.^[^ ^58^ ^]^ In this study, we fused a biotin acceptor peptide (BAP) with NOS1. The Biotinylated NOS1‐BAP bound to streptavidin‐coated QD625 (SA‐QD625), resulting in the formation of NOS1‐BAP‐QD complexes. Under the action of an NLS, NOS1‐BAP‐QDs were transported into the nucleus (**Figure** **7**A,B; Figure S17B, Supporting Information). Only SA‐QD625 were transfected alone or NOS1N‐NLS‐BAP without biotinylated did not result in the entry of SA‐QD625 into the cell nucleus (Figure S17A,B, Supporting Information). Additionally, the multivalent interactions between NOS1N molecules allowed NOS1N‐QD625 to selectively target and enter NOS1N condensates (Figure 7B,C). When QDs are immobilized or diffused in solution, the fluorescence brightness of individual QDs is similar, but surface deposition results in brighter clusters, indicating the presence of aggregates.^[^ ^59^ ^]^ Single‐particle tracking was performed at a frame rate of 100 ms (Movies S1 and S2, Supporting Information). The tracking duration was limited to the time for which the particles remained within a single focal plane because the required acquisition rate did not allow z‐stack collection. The total tracking time was 10 s. We analyzed thousands of motion traces and extracted the mean square displacement (MSD) and effective diffusion coefficient (Deff) within a 10 s time frame. Figure 7D and E represent the motion trajectories and velocities of the QDs within the condensates.

We compared the MSD and Deff values of the QDs inside NOS1N condensates with those inside the nucleus (Figure 7F,G). The MSD and Deff values of the QDs inside the NOS1N condensates were lower than those outside the condensates, suggesting that the motion of the QDs inside the NOS1 condensates was restricted, whereas the motion outside the condensates followed simple diffusion. We examined the MSD curves and found that the motion of QDs within the condensates was subdiffusive. This subdiffusive motion can be attributed to local confinement in the crowded environment and/or interactions between the tracking particles and the surroundings.

## 3 Discussion

### 3.1 MRC: A Tool for Visually Studying PPIs and Expands the Current Scope of PPI Research

Colocalization‐based PPI studies are limited by image resolution. MRC recruits interacting proteins to the same location, resulting in precise colocalization signals. It is easy to operate, requires moderate instrument requirements, and has a short experimental cycle for PPIs studies in live cells. More than that, MRC has other advantages in studying PPIs. First, validating whether proteins interact directly with each other. MRC is somewhat reliant on the abundance of nuclear proteins and requires their substantial overexpression. This could be advantageous because it prevents interference from freely diffusing target proteins in the nucleus when studying nuclear protein interactions. Second, MRC utilizes multichannel fluorescence spectroscopy to validate multi‐protein interactions in live cells. Multi‐protein interactions within complexes were validated in this study, including SCF, GPCRs, and NOS1‐PSD95‐NOS1AP ternary complexes. Third, MRC can assess the binding capacity of proteins within complexes by calculating their respective colocalization percentages and SNR ratios. For example, GNB1 showed stronger binding to GNG2 than GNB2, and RBX1 enhanced NEDD8–CUL1 interaction, consistent with previous findings on the RBX1 RING domain's role in NEDD8 transfer.^[^ ^60^ ^]^ However, since many factors can influence the recruitment of colocalization, it provides only a general evaluation of binding ability and cannot be fully quantitative. For example, imaging conditions (e.g., exposure time, illumination) can affect the SNR of spots, and their impact was not specifically evaluated in this study. To reduce potential errors, multiple repeats are necessary. Therefore, while this method serves as a preliminary assessment and cannot be used for fully quantitative measurements, it remains a simple and intuitive approach for evaluating PPI binding capability.

Compared to the previous F3H method, this approach achieves higher positive detection rates by recruiting more proteins through a dual recruitment strategy, facilitating the observation of multiprotein complexes. In this study, multiple protein interactions and interactions among multiprotein complexes were successfully validated. Additionally, this method offers greater flexibility in the selection of chromosomal loci and repeat sequences. By simply adding sgRNA, more repeat sequences can be generated to recruit additional bait proteins. This technique can be broadly applied to various cell types, including plants, without requiring additional modifications. Furthermore, the application of MRC has been expanded in this study. For example, when combined with BiFC, it enables the simultaneous observation of interactions among more than three proteins, which was not achievable with previous methods. Moreover, when integrated with phase separation, this approach can recruit more bait proteins, enhance colocalization signals, and improve detection sensitivity, making it particularly useful for validating weak protein interactions. These advantages highlight the strong expandability of MRC and its potential for further development in the future. We systematically compared several techniques for visualizing PPIs in live cells; detailed comparisons are provided in Tables S4 and S5 (Supporting Information). Future developments may focus on quantitative modeling or real‐time monitoring of binding via SNR dynamics, with applications in protein interaction kinetics and inhibitor screening.

### 3.2 MRC Can Study the Properties of Phase‐Separating Proteins in Live Cells and As a Multifunctional Tool Can Also Investigate the Relationship between PPIs and Condensates Formation

Many proteins or protein domains have the potential to undergo phase separation, but their propensity varies. Some proteins, even when highly expressed, may not form distinct condensate structures. Systematic assessment of the phase separation capabilities of proteins remains challenging.^[^ ^27^, ^28^ ^]^ In this study, we used the MRC method to induce phase separation of the target proteins in live cells. Through this approach, we identified the phase separation capability within the NOS1 1–235 amino acid region and validated its interaction with PSD95. Both the PDZ and IDR of NOS1 jointly facilitate classical intracellular phase separation, resulting in distinct liquid‐like features of intracellular condensates. This method also enabled comparison of condensate‐forming abilities across proteins, for instance, FUSN readily nucleates condensates, while NOS1N requires additional recruitment to genomic loci to form a greater number of condensates.

One important application of using MRC to study phase separation is integrating its analysis of protein complex PPI characteristics to investigate the mechanism of condensate formation. For example, in this study, by examining the complex formed through the interaction between NOS1 and PSD95, it was found that the interaction between PSD95 and NOS1 promotes the formation of NOS1N condensates. The interaction between PSD95 and NOS1N may introduce multivalency, thereby augmenting multivalency and consequently enhancing NOS1N's propensity for phase separation. In many cases, many proteins in the cellular environment cannot undergo phase separation alone under physiological conditions. They require co‐interactions with multiple proteins or forms, such as protein/RNA or DNA complexes, to collectively surpass a threshold and initiate phase separation.^[^ ^61^, ^62^, ^63^ ^]^ For example, phase separation of SynGAP and PSD‐95 complex requires multivalent interactions between SynGAP and PSD‐95, PSD95‐PSG (aa R306‐L721) alone cannot independently form a condensate phase.^[^ ^63^ ^]^ Therefore, MRC enables the simultaneous exploration of protein–protein interactions with complex and assessment of the interaction partners’ contribution to phase separation.

### 3.3 Using MRC to Rapidly Evaluate Condensate Formation Potential of Proteins or Domains in Live Cells

Through the application of SunTag and genomic repeat locus amplification, highly localized concentration of target proteins can be achieved, which helps identify proteins with potential phase separation capability. The MRC system enables intuitive and efficient preliminary screening and evaluation of the phase separation condensate‐forming potential of specific proteins or domains in living cells. In this study, we observed that the PDZ domains of APBA, GRIP, and MPDZ formed condensate‐like structures in cells. While different proteins show varying degrees of multivalent self‐aggregation within their PDZ domains.^[^ ^48^ ^]^ However, it is worth noting that MRC validated phase separation potential using SunTag and genomic repeat sequence amplification, as well as overexpression of the target protein, which may amplify the phase separation capability of the target proteins. The results would not necessarily mean that the protein undergoes phase separation in its physiological state. Moreover, it cannot immediately distinguish whether the condensate is a droplet or a gel, requiring further experiments, such as FRAP, to discern the properties of these condensates.

### 3.4 Utilizing MRC for Generating Multiple Condensates in Designated Spatial Loci

Previous studies showed that nuclear condensates may not mix due to surface tension differences, resulting in layered multiphase condensates within the nucleus.^[^ ^64^ ^]^ Using MRC, we confirmed interactions between FUSN and TAF15N, which formed merged rather than separate condensates. In contrast, non‐interacting FUSN and NOS1N formed adjacent but distinct condensates that did not fuse upon growth. This tool facilitates the study of how the interactions between different condensates affect their growth and phase behavior. Further efforts will be made to generate multiple phase‐separated condensates at additional specified target sites, enabling further investigation of localized signal amplification or response, local signal transduction, subcellular functional compartmentalization, cell architecture, and morphology maintenance. Notably, current MRC‐induced condensates are nuclear; further development could enable induction in the cytoplasm or other organelles.

### 3.5 MRC Can be Used for Programmable Synthesis of Condensates within Living Cells

We integrated the optogenetic tool into the MRC phase separation system, allowing the programmable recruitment of non‐phase separating proteins into engineered condensates through light‐induced dimerization. This dense aggregation enhances coaggregation and improves colocalization efficiency for PPIs studies. However, the amplification effect complicates distinguishing direct from indirect interactions. Some studies have employed engineering methods to synthesize condensates in eukaryotic cells and bacteria, and the manipulation of cellular behavior is possible.^[^ ^62^, ^65^, ^66^, ^67^, ^68^ ^]^ In the current approach, MRC enables non‐phase‐separating proteins to be recruited into programmable condensates within living cells. Additionally, the biogenic condensates created within cells are programmable with MRC, allowing manipulation of the physical characteristics of the target protein using condensates of different natures. These programmable condensates can tune target protein behavior, offering promising applications in synthetic biology—such as regulating enzyme activity, isolating pathways, buffering translation, scaffolding interactions, and drug discovery.^[^ ^24^, ^69^, ^70^, ^71^, ^72^, ^73^ ^]^

### 3.6 MRC is Used to Probe the Physical Properties of Condensates in Live Cells

Previous studies have employed genetically encoded nanoparticles (GEMS) as fluorescent probes to determine the size of cellular solute microrheology in the cytosol.^[^ ^74^ ^]^ Here, we used quantum dots (QDs), which enter the nucleus only when bound to NLS‐tagged proteins,^[^ ^75^ ^]^ and access condensates via multivalent protein interactions. Due to the high photostability of quantum dots, we can theoretically perform long‐term single‐particle tracking of the movement of phase‐separating proteins within condensates in live cells with high spatiotemporal resolution. This approach allows the assessment of viscosity, crowding, and monitoring of condensate density in various condensates as rheological probes. Upon it, we would be able to further ascertain whether these specific properties have any impact on the functionality.

In conclusion: The method developed in this study can serve as a multifunctional and versatile tool for studying various multiple protein interactions and biomolecular condensates in living cells.

## 4 Experimental Section

### Plasmid Construction

To construct plasmids used for stable transfection of cell lines, the following procedures were employed. The study synthesized a nuclear localization signal (NLS) sequence with the termination codon TAA and introduced a NotI restriction enzyme site into the pHRdSV40‐dCas9‐10xGCN4_v4‐P2A‐BFP plasmid (Addgene #60903). Subsequently, a 10xGCN4 sequence was inserted into this fragment, leading to the premature termination of expression after mTagBFP2. The pHRdSV40‐NLS‐dCas9‐24xGCN4_v4‐NLS‐P2A‐BFP‐dWPRE plasmid (Addgene #60910) was constructed by removing the 24xGCN and mTagBFP2 portions using BamHI and PacI digestion. Subsequently, the 24xGCN4 fragment was amplified and cloned using the Golden Gate cloning method. This resulted in the generation of pHRdSV40‐NLS‐dCas9‐24xGCN4_v4‐NLS‐P2A‐dWPRE and pHRdSV40‐NLS‐dCas9‐10xGCN4_v4‐NLS‐P2A‐dWPRE. To clone dCas9‐2xGCN4, dCas9‐10xGCN4, and dCas9‐24xGCN4 fragments into the transpon vector, these fragments were amplified by PCR and inserted into the EcoRI‐digested Transpon vector (The GFP sequence was removed from the vector). This yielded Trans‐SunTag2x, Trans‐SunTag10x, and Trans‐SunTag24x constructs.

For a series of target genes anchored to genomic loci and carrying scFv‐sfGFP vectors, the procedure involved initial RT‐PCR amplification of the desired target genes from either 293T cells or brain glioma cell cDNA. Taking the acquisition of pHR‐scFv‐sfGFP‐CUL1 as an example, golden gate cloning was used to introduce the CUL1 fragment into the pHR‐scFv‐GCN4‐sfGFP‐GB1‐NLS‐dWPRE vector after SpeI digestion. For target genes anchored to genomic loci and carrying scFv‐mCherry vectors, the mCherry sequence was first amplified, and BamHI and RsrII sites were introduced to obtain the pHR‐scFv‐GCN4‐mCherry‐GB1‐NLS‐dWPRE vector. Subsequently, the target gene was inserted into the SpeI‐digested sites.

For target genes with NLS‐mCherry or NLS‐mTagBFP2 vectors, the MCP and 3XBFP sequences in the pHAGE‐EFS‐MCP‐3XBFPnls vector (#75 384; Addgene) were initially replaced with mCherry and mTagBFP2, respectively, using EcoRI and BamHI sites. A linker segment and KpnI restriction site were introduced, resulting in the pHAGE‐EFS‐mCherry vector. Subsequently, these genes were inserted into the KpnI cloning site. After amplifying the cDNA products of various genes, golden gate cloning was employed to insert them into KpnI‐digested pHAGE‐EFS‐mCherry or pHAGE‐EFS‐mTagBFP2 vectors. This methodology resulted in the development of pHAGE‐EFS‐RBX1‐mCherry and pHAGE‐EFS‐SKP1‐mTagBFP2.

sgRNA vector construction involved the synthesis of sgRNA sequences for *MUC4^‐272 kb^* and *MUC4^‐272kb‐42^*, which were inserted into the pX330‐spsgRNA‐TER3G‐1nls‐spdCas9‐2nls‐mCherry‐10tag‐1nls vector. Subsequently, the U6‐sgRNA‐scaffold fragment was amplified and introduced into the pLH‐sgRNA1‐2XMS2 (Addgene #75 389) vector using the SanDI and EcoRI restriction sites.

The construction of TetON‐SKP1‐mCherry included amplification of the SFFV‐TETON sequence from the pHAGE‐sffv‐TETON3G‐CMV‐PURO vector and the TER3G promoter region from the pX330‐spsgRNA‐TER3G‐1nls‐spdCas9‐2nls‐mCherry‐10tag‐1nls vector. These two fragments were then connected using golden gate cloning to generate the pHAGE‐EFS‐SKP1‐mCherry vector, and by replacing the original EF‐1α core promoter with NheI and MluI restriction sites, the pHAGE‐TER3G‐EFS‐SKP1‐mCherry vector was obtained.

For constructing MoonTag‐related vectors, the Nb‐gp41 sequence was amplified from the Nb‐gp41‐Halo vector and inserted into the XhoI restriction site of FUSN‐mCherry and TAF15‐mCherry vectors to yield FUSN‐mCherry‐Nb‐gp41 and TAF15‐mCherry‐Nb‐gp41, respectively. The Nb‐gp41 sequence was amplified and inserted at the KpnI site of the pHAGE‐EFS‐mCherry vector to generate pHAGE‐EFS‐Nb‐gp41‐mCherry. Subsequently, FUSN and TAF15 were introduced into pHAGE‐EFS‐Nb‐gp41‐mCherry via XhoI to produce pHAGE‐EFS‐Nb‐gp41‐mCherry‐FUSN and pHAGE‐EFS‐Nb‐gp41‐mCherry‐TAF15 vectors, respectively. Vectors carrying SadCas9 and MoonTag‐related components were constructed by amplifying the SadCas9‐mCherry sequence from the SadCas9‐mCherry vector, inserting it into the KpnI site of the RBX1‐mCherry vector, replacing RBX1, yielding the pHAGE‐SadCas9‐mCherry vector. Subsequently, the 10xgp41‐peptides and 24xgp41‐peptides sequences were amplified from the 24xMoonTag vector and inserted into the pHAGE‐SadCas9‐mCherry vector using EcoRI and XhoI to generate the pHAGE‐SadCas9‐mCherry‐MoonTag24x vector. Finally, the U6‐MUC4E‐scaffold sequence was amplified and inserted into the NheI site to obtain the pHAGE‐MUC4E‐dSaCas9‐MoonTag24x vector.

To construct optogenetics‐related vectors, sspB, iLID, CRY2, and CIBN sequences were synthesized by Shanghai Biotechnology Corporation. The NOS1N‐scFv‐sfGFP‐iLID vector was obtained by inserting iLID into the RsrII restriction site of NOS1N‐scFv‐sfGFP and introducing iLID into the BamHI‐ and RsrII‐digested NOS1N‐scFv‐sfGFP vectors removed the original sfGFP sequence, resulting in the NOS1N‐scFv‐sfGFP‐iLID vector. Similarly, the NOS1N‐scFv‐CIBN vector was generated. pHAGE‐EFS‐SKP1‐mCherry‐sspB and pHAGE‐EFS‐SKP1‐mCherry‐CRY2 fragments were inserted into the XhoI sites of the pHAGE‐EFS‐SKP1‐mCherry vector. Introducing BAP into NOS1N‐mTagBFP2 to obtain NOS1N‐mTagBFP2‐BAP.

### Cell Culture, Transfection, and Stable Cell Lines

The morphologies of the HeLa and human HEK293T cells were validated. HEK 293T and HeLa cells were cultured in Dulbecco's modified Eagle's medium (DMEM, HyClone) supplemented with 10% Fetal Bovine Serum (FBS, Gibco) at 37 °C and 5% CO2. Cells were transfected in Opti‐MEM (Gibco, Invitrogen) using Lipofectamine 3000 (Invitrogen, Carlsbad, CA, USA) according to the manufacturer's protocol.

To establish stable cell lines, HEK 293T cells were seeded in six‐well plates. The following day, cells at ≈70% confluency were transfected with 1.5 µg of Trans‐SunTag2x, Trans‐SunTag10x, or Trans‐SunTag24x, as well as 0.5 µg of pCAG‐hyPBase, using Lipofectamine 3000. Positive clones were selected using puromycin (4 µg mL^−1^) for ≈2 weeks. Cells were collected, and western blot analysis using anti‐HA antibodies was performed to confirm the expression of SunTag2x, SunTag10x or Trans‐SunTag24x in the established stable cell lines.

For the transient transfection experiments prior to imaging, the assembly process of the CRISPR‐dCas9/SunTag system is taken as an example. The following plasmids were co‐transfected into pre‐established SunTag10x‐dCas9‐HEK293T or SunTag24x‐dCas9‐HEK293T cells: pLH‐sgRNA‐MUC4‐272 kb (encoding the sgRNA targeting the genomic locus), pHR‐scFv‐GCN4‐sfGFP‐GB1‐protein1‐NLS‐dWPRE (encoding the bait protein), and pHAGE‐protein2‐mCherry‐NLS or pHAGE‐protein3‐mTagBFP2‐NLS (encoding the interacting protein), with 500 ng of each plasmid. Transfection was performed using Lipofectamine 3000 reagent according to the manufacturer's instructions. After transfection, the cells were incubated at 37 °C, and imaging was performed 24 h later.

### Protein Expression and Purification

Amplified NOS1N‐sfGFP and PSD95‐mCherry fragments were inserted into the EcoRI‐digested site of the pET28a+ vector. The recombinant plasmids, pET28a‐NOS1N‐sfGFP and pET28a‐PSD95‐mCherry, were transformed into Escherichia coli Rosetta (DE3) cells for protein expression. Bacterial cultures were grown in LB medium at 37 °C with shaking until they reached the logarithmic growth phase. Induction was carried out at 16 °C for 12 h using 0.5 mm isopropyl β‐D‐thiogalactoside (IPTG).

Cells were collected by centrifugation, resuspended in 15 mL of Buffer A (20 mm Tris, pH 7.5, 500 mm NaCl, 10% v/v glycerol, and 1 mm phenylmethanesulfonyl fluoride (PMSF)), and lysed by sonication. The lysate was centrifuged at 35 000 rpm for 60 min, and the supernatant was collected. The supernatant was loaded onto a pre‐equilibrated 4 mL HisTrap HP column (GE Healthcare) within a polypropylene column (QIAGEN, 34 964). The protein was eluted with buffer A or B supplemented with 500 mm imidazole.

Subsequently, size exclusion chromatography was performed using a Superdex‐200 column on an AKTA purifier (GE Healthcare Life Sciences, Boston, MA, USA) to further purify the protein. The purified protein was concentrated to 2 mL using an Amicon Ultra centrifugal filter unit (Millipore, UFC901096). Protein samples were analyzed using SDS‐PAGE to confirm their purity and concentration. The protein concentration was determined using an enhanced BCA protein assay kit (P0010, Beyotime Biotechnology, China).

### In Vitro Induction of Phase Separation

All purified NOS1N‐sfGFP or PSD95‐mCherry fusion proteins were concentrated to the same volume (50 µL) at the same concentration (5 µmol L^−1^). These concentrated proteins were individually or combined in 1.5 mL centrifuge tubes, followed by the immediate addition of 10% (v/v) PEG‐2000 as a crowding agent. Samples were taken at 0, 2, 8, and 16 h, and 5 µL of each sample was spotted onto glass slides for imaging using the imaging channels of a Cytation 3 instrument (BioTek Instruments, USA).

### FRAP Assay

Fluorescence Recovery After Photobleaching (FRAP) was conducted on an inverted laser scanning confocal microscope (Zeiss, LSM980 AxioObserver.Z1/7), equipped with a motorized stage, a heated stage, and a full incubation chamber maintaining 37 °C and 5% CO2. The 488 nm laser was used for the FRAP measurements. Images were acquired using a 63x Plan‐Apochromat NA1.40 oil immersion objective under the control of Zeiss Zen software. Partial photobleaching of the viral protein‐DNA puncta was performed using 100% laser power. Intensity trajectories were analyzed using Imaris 9.1 software (Bitplane AG, Oxford Instruments, Zurich, Switzerland). Drift effects were minimized by subtracting the intensity trace of the control condensate from that of the photobleached condensate. The intensity trace of each condensate was standardized between the pre‐bleach and post‐bleach frames. The averages and standard deviations of the recovery traces were calculated.

To calculate the normalized fractional fluorescence recovery, the intensity at the first post‐bleach time point (i.e., *t* = 0) was set to zero, yielding:

(1) $$R_{frac}\left(t\right)=\frac{R_{norm}\left(t\right)-R_{norm}\left(0\right)}{1-R_{norm}\left(0\right)}$$

The resulting data were then fitted using a single exponential recovery function:

(2) $$R_{frac}\left(t\right)=A\left.\left(1-e^{-t/\tau }\right.\right)$$

where τ is the time constant of fluorescence recovery, and A represents the mobile fraction of the fluorescent probe within the region of interest (ROI). The percentage recovery in the ROI was calculated as:

(3) %Rrecover=A×100

### Fluorescent Imaging

Live cell imaging was performed using a Delta Vision OMX V3 imaging system (GE Healthcare, USA). The system was equipped with a 100 × 1.4 NA oil immersion objective (Olympus, UPlanSAPO) and solid‐state multimode lasers emitting at 405 nm (for mTagBFP2), 488 nm (for sfGFP), and 561 nm (for mCherry). Images were captured using an electron‐multiplying, charge‐coupled device camera (Evolve 512 × 512; Photometrics). The microscope was typically calibrated using 100 nm fluorescent beads to calculate the lateral and axial limits of image resolution. Image stacks were reconstructed using SoftWoRx 6.1.1 software (GE Healthcare, USA) and further processed to obtain maximum intensity projection images.

### Photoactivation

In the Delta Vision OMX V3 imaging system, photoactivation was performed under the following conditions: using a 488 nm laser with a power density of 100 mW cm^−^^2^ and an exposure time of 10 milliseconds, repeated every 15 s for a total of 5 min.

### Imaging Data Analysis

The relative nuclear fluorescence intensity was calculated for each cell by normalizing the nuclear sfGFP fluorescence intensity values at each time point to the initial intensity value at the start of the experiment (time point 0). Signal‐to‐noise ratio (SNR) was defined as the ratio of fluorescent signal intensity to background noise power, as shown in the following formula: SNR = (P_signal)/(P_noise) = (Maximum intensity of GFP spot – Mean intensity of background fluorescence signal)/(Standard deviation of background fluorescence signal), where a circular region with a diameter of 3–4 µm and centered at a spot (excluding spots with labeled signals) was selected as the background.

The image stacks were reconstructed using SoftWoRx 6.1.1 (GE Healthcare, USA) and further processed to obtain maximum intensity projection images. The areas and diameters of the spots in the stacked images were calculated using Imaris 9.1 surface analysis. A surface function was employed to automatically identify spots in the stack images, with a threshold set to effectively eliminate background noise.

The fluorescence intensity along the Z‐axis of the original image stacks was determined using ImageJ software, and the fluorescence values were entered into Excel. To obtain more accurate 3D images along the Z‐axis, images were captured at frame intervals of 125 nm along the Z‐axis to generate Z‐stack images. The original image format was opened using Imaris to obtain the 3D images. Colocalization analysis was carried out using the Coloc 2 plugins in Fiji/ImageJ

To evaluate the morphology of protein condensates, circularity analysis was performed using ImageJ. Fluorescence images were acquired under consistent imaging conditions. For each condition, five condensates were randomly selected for analysis (n = 5). The outlines of condensates were automatically detected using ImageJ's “Analyze Particles” function, which also calculated their circularity values. A circularity value of 1.0 indicates a perfect circle, while values approaching 0.0 reflect increasingly elongated or irregular shapes.

### Doxycycline Induction of Expression

Cells were transfected with the corresponding plasmids and tetracycline‐inducible (TETON3G) vectors. After 24 h of cultivation to allow for TETON3G expression, induction was initiated by adding 1 µg ml^−1^ of doxycycline (Sigma–Aldrich). The cells were imaged 24 h after doxycycline induction.

### ZL006 Drug Treatments

ZL006 (MedChem Express) was dissolved in 10% dimethyl sulfoxide (DMSO). Following pre‐seeding of cell culture dishes with cells at 70% confluency, NOS1N and PSD95 plasmids were transfected into glass‐bottom dishes. Subsequently, ZL006 was added to achieve a final concentration of 100 µm. Imaging results were obtained after 24 h.

### Transfection and Fusion of QDs to Target Protein

To study the dynamic properties of phase‐separated condensates using the MRC system combined with quantum dot labeling, the following example plasmids and reagents were used: pHR‐scFv‐GCN4‐sfGFP‐NOS1N, NOS1N‐mTagBFP2‐BAP, pcDNA3.1(+)‐BirA (for intracellular biotinylation), pLH‐sgRNA‐MUC4‐272 kb, and Streptavidin‐conjugated QD625 (SA‐QD625, Thermo Fisher Scientific, 10 µm). HEK293T cells were seeded in 35 mm culture dishes and transfected 24 h later using Lipofectamine 3000 with the following amounts: 500 ng pcDNA3.1(+)‐BirA, 500 ng each of pHR‐scFv‐GCN4‐sfGFP‐NOS1N, NOS1N‐mTagBFP2‐BAP, pLH‐sgRNA‐MUC4‐272 kb, and 10 µL SA‐QD625 (10 µm). Two hours after transfection, 50 µm biotin was added to the culture medium to facilitate BirA‐mediated biotinylation of the BAP tag.

### QDs Paraspeckle Movement Tracking Analysis

Z‐axis images were initially captured using OMX to visualize the encapsulation of the QDs within the condensate structure. Upon confirming the presence of QDs within the condensates, wide‐field mode imaging was conducted at a rate of 100 ms frames per second with a 10 s interval between images. The raw image files were imported into Imaris software to generate motion trajectory maps and extract various parameters from the particle trajectories. For each trajectory, the time‐averaged mean squared displacement (MSD) using the Imaris MSD plug‐in. MSD curves were fitted using the following equation: MSD = 4D_eff_ Δt, where D_eff_ represents the effective diffusion coefficient of the particles.

### RNA Isolation and cDNA Synthesis

HEK293T cells or brain glioma cells RNA extraction was performed using Trizol Reagent (Invitrogen Carlsbad, CA) following the manufacturer's instructions. cDNA was synthesized using the TransScript One‐Step gDNA Removal and cDNA Synthesis SuperMix (TransGen Biotech, Beijing, China).

### Protein Disorder Prediction

Protein IDRs prediction was performed using the PONDR@webtool^[^ ^76^ ^]^ with the VSL2 algorithm (http://www.pondr.com/).

### Statistical Analysis

Data were analyzed using GraphPad Prism 8. Comparisons between two groups were performed using an unpaired Student's t‐test. For comparisons among more than two groups, one‐way analysis of variance (ANOVA) followed by Tukey's Honestly Significant Difference (HSD) post hoc test was used. Assumptions of normality and homogeneity of variances were tested and met for all ANOVA applications. No data transformation or outlier exclusion was performed unless otherwise noted. Statistical significance was set at *p* < 0.05. Data are presented as mean ± standard deviation (SD). Sample size (n) is indicated in the respective figure captions and represents data from at least three independent experiments unless otherwise stated.

## Conflict of Interest

The authors declare no conflict of interest.

## Author Contributions

L.P. and X.E.Z. conceptualized the study. L.P. performed the experiments and data analyses. L.P. and X.E.Z. completed the manuscript. Y.H. assisted with imaging data analysis and plasmid construction. Y.Z. assisted with manuscript revision. J.Y.T. aided in protein purification. L.S. and B.P. assisted with plasmid construction. M.L. and D.B.W. contributed helpful suggestions for the experiments. X.E.Z. supervised the study.

## Supporting information



Supporting Information

Supplemental Movie 1

Supplemental Movie 2

## Data Availability

The data that support the findings of this study are available from the corresponding author upon reasonable request.
