# Supplementary material for: A Molecular Recruitment Colocalization Platform for Visualizing Multi‐Protein Interactions and Engineering Biomolecular Condensates in Living Cells
Source: Adv Sci (Weinh). 2025 Jul 22;12(37):e05455. doi: 10.1002/advs.202505455 (PMC12499469; doi:10.1002/advs.202505455)
Supplement: Supplementary file 1 — Supporting Information [file ADVS-12-e05455-s003.docx]

**Supplementary Information**

**A Molecular Recruitment Colocalization Platform for Visualizing Multi-protein Interactions and Engineering Biomolecular Condensates in Living Cells**


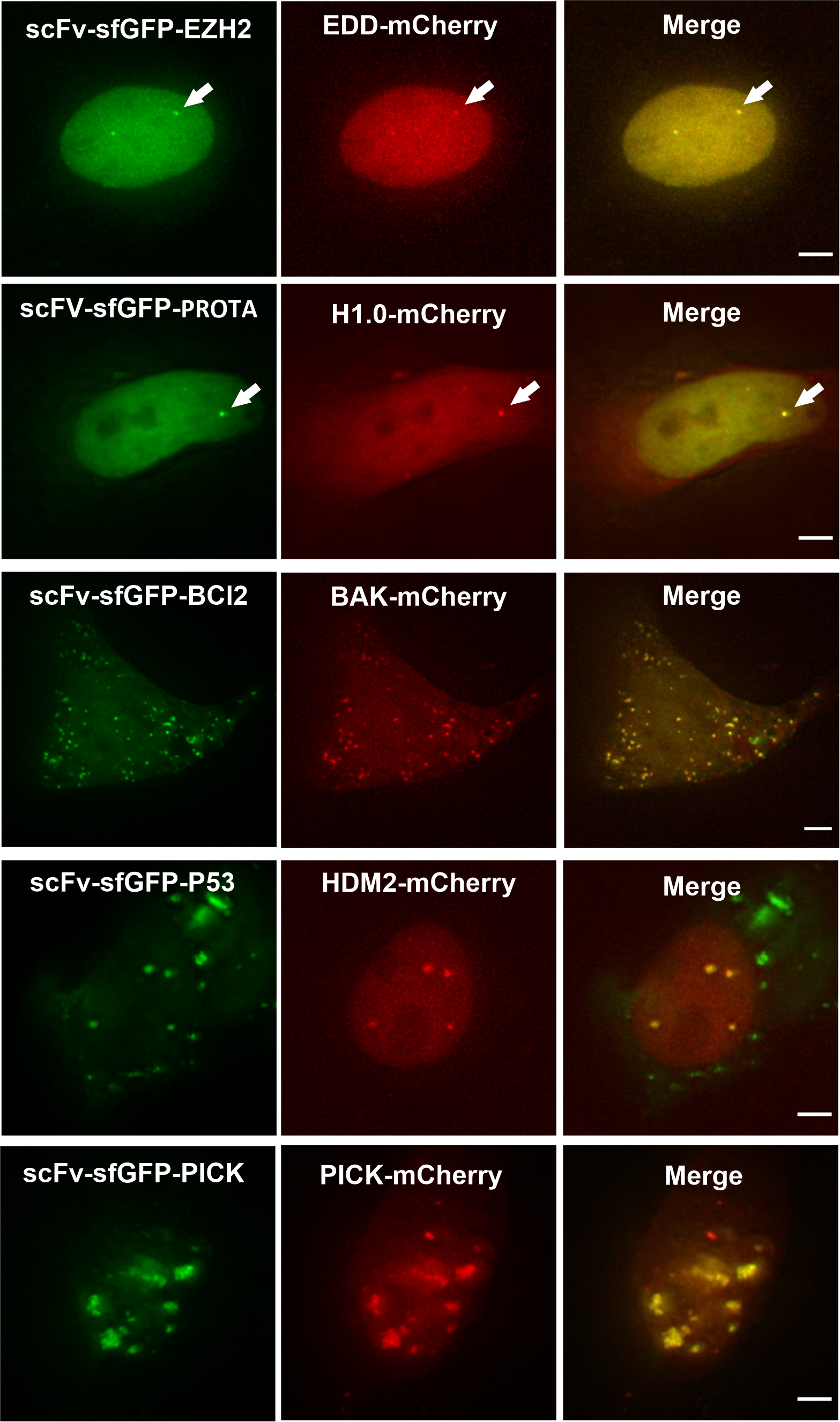


**Supplementary Figure 1. Live-cell imaging of protein interactions validated by MRC.** Positive colocalization points are formed between interacting proteins. All Scale bar, 3 μm. Due to self-aggregation, BCL2, P53, and PICK form multiple points within the nucleus.


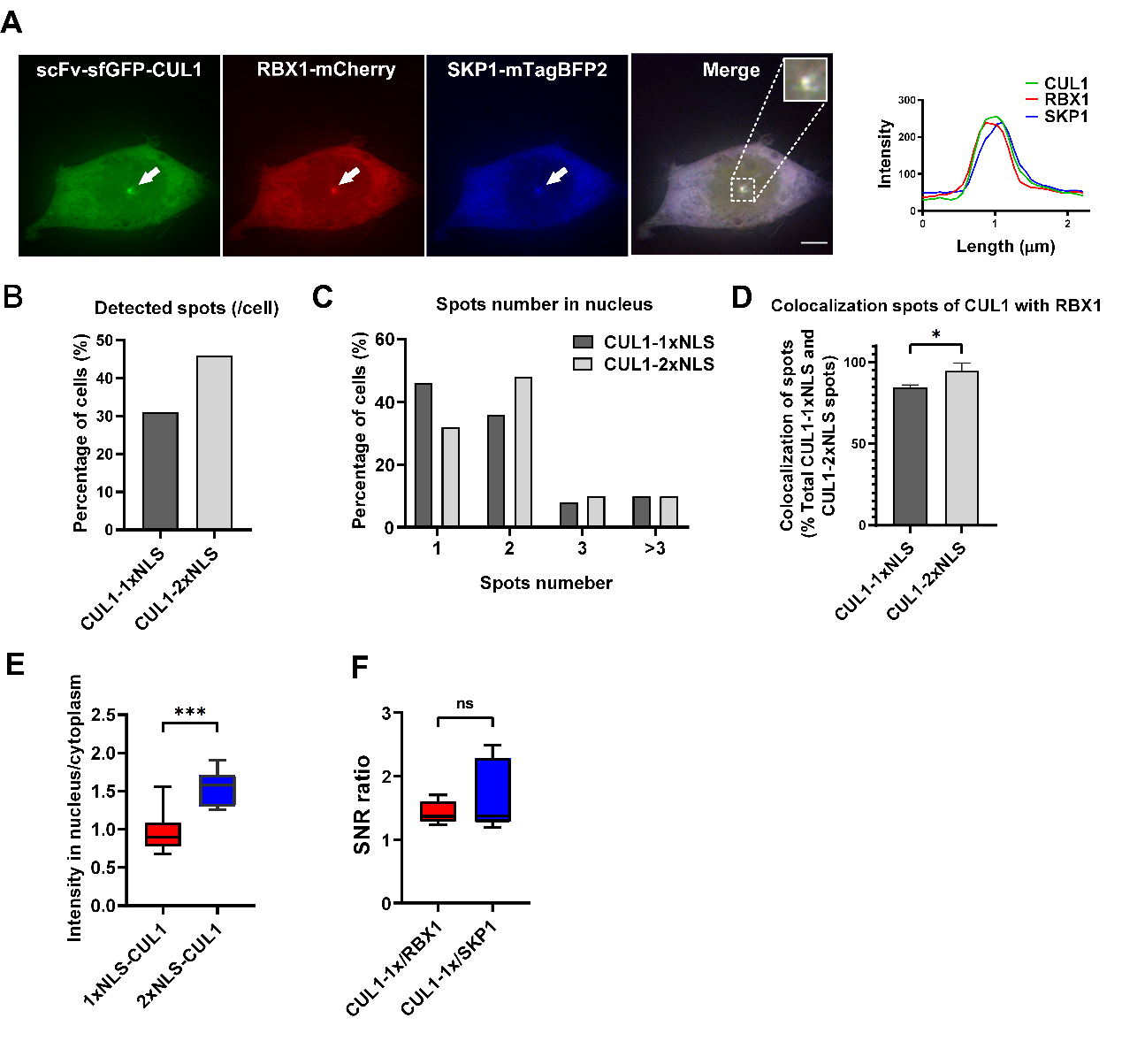


**Supplementary Figure 2. Influence of 1xNLS and 2xNLS on nuclear import and recruitment of interacting proteins by CUL1.**

**(A)** Representative images illustrate the recruitment of 1xNLS-scFv-sfGFP-CUL1 in cells and its colocalization with RBX1-mCherry and SKP1-mTagBFP2. Corresponding constructs were co-transfected into SunTag10x-dCas9-HEK293T cell lines. Dashed boxes show enlarged condensate images, and the right panels correspond to the fluorescence intensity line profiles of the enlarged images. All scale bars, 3 μm.

**(B)** The proportion of cells with successfully formed green spots by recruiting 1xNLS-scFv-sfGFP-CUL1 and 2xNLS-scFv-sfGFP-CUL1, n = 100 cells.

**(C)** The distribution percentage of the number of green spots of 1xNLS-scFv-sfGFP-CUL1 and 2xNLS-scFv-sfGFP-CUL1, n = 100 cells.

**(D)** Percentage of colocalized spots of 1xNLS-scFv-sfGFP-CUL1 and 2xNLS-scFv-sfGFP-CUL1 with RBX1. Graphs show the mean ± SD with three biological replicates, and each replicate contains 15 cells. Comparison between the two groups was performed using a t-test. “*” indicates a significant difference (*p* < 0.05).

**(E)** Ratio of nuclear to cytoplasmic fluorescence for 1xNLS-scFv-sfGFP-CUL1 and 2xNLS-scFv-sfGFP-CUL1, with a total of 5 cells. Comparison between the two groups was performed using a t-test. “***” indicates a significant difference (*p* < 0.001).

**(F)** The ratio of SNR values among 1xNLS-scFv-sfGFP-CUL1 (CUL1-1x), RBX1, and SKP1, n = 5 co-localized spots. Comparison between the two groups was performed using a t-test. “*” indicates a significant difference (*p* < 0.05).


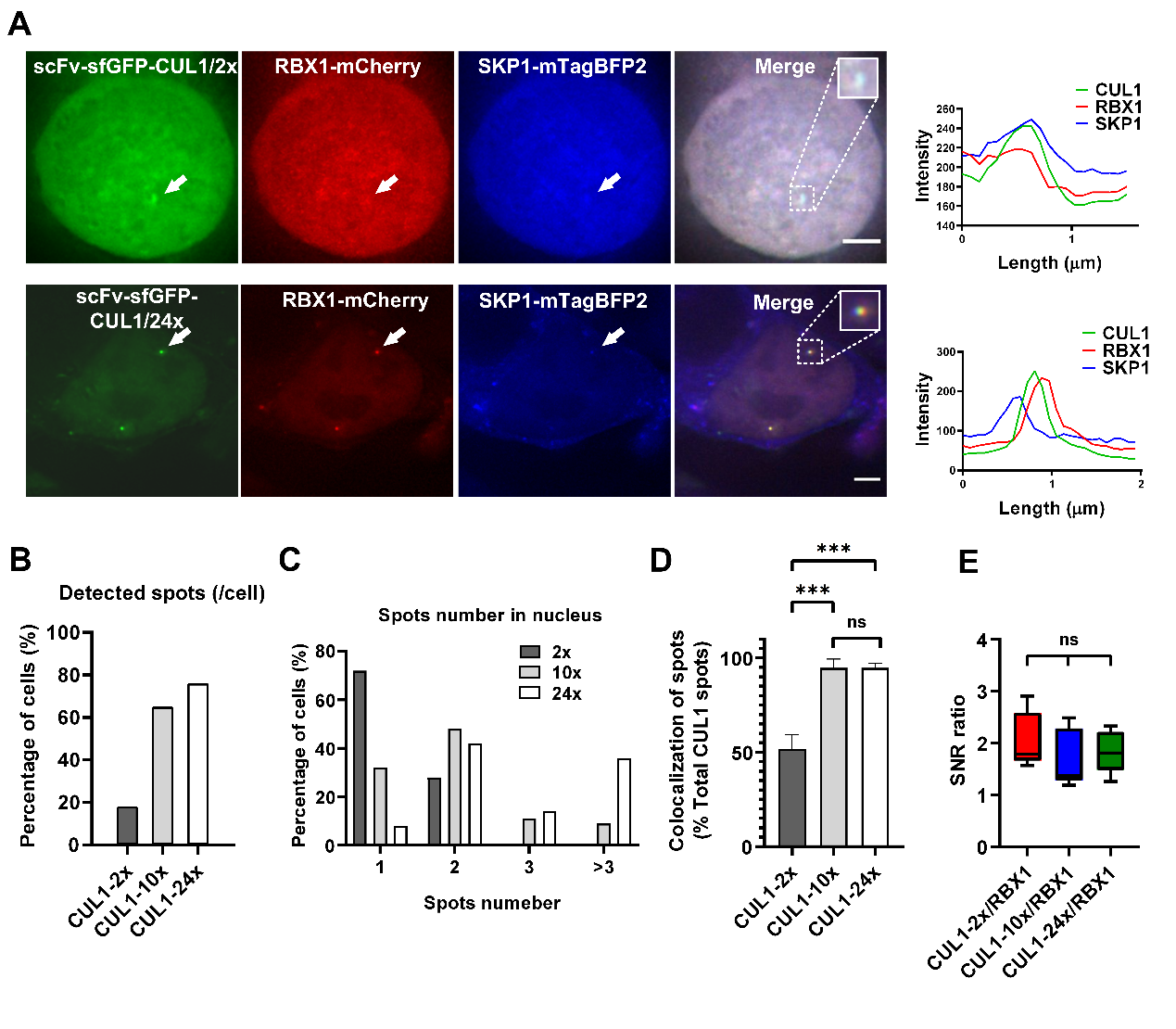


**Supplementary Figure 3. Comparison of positivity rates for CUL1/2xGCN4, CUL1/10xGCN4, and CUL1/24xGCN4.**

**(A)** Comparison of live cell images showing the interaction of CUL1/2xGCN4, CUL1/10xGCN4, and CUL1/24xGCN4 with RBX1 and SKP1. Corresponding constructs were co-transfected into respective SunTag2x-dCas9-HEK293T, SunTag10x-dCas9-HEK293T, and SunTag24x-dCas9-HEK293T cell lines. Dashed boxes show enlarged condensate images, and the right panels correspond to the fluorescence intensity line profiles of the enlarged images. All Scale bars, 3 μm.

**(B)** The proportion of cells with successfully formed spots by recruiting CUL1/2xGCN4, CUL1/10xGCN4, and CUL1/24xGCN4, respectively.

**(C)** Distribution of the percentage of cells with successfully recruited green spots by CUL1/2xGCN4, CUL1/10xGCN4, and CUL1/24xGCN4, with n = 100 cells.

**(D)** Percentage of cells with successful colocalization of CUL1/2xGCN4, CUL1/10xGCN4, and CUL1/24xGCN4 with RBX1. Graphs show the mean ± SD with three biological replicates and each replicate contains 15 cells. Group comparisons were performed using Tukey’s HSD test. “*” *p* < 0.05. “ns” no significance, *p* > 0.05. “***” indicates a significant difference (*p* < 0.001).

**(E)** The ratio of SNR values between CUL1/2xGCN4, CUL1/10xGCN4, and CUL1/24xGCN4 with RBX1, n = 5 co-localized spots. Group comparisons were performed using Tukey’s HSD test. “ns” no significance, *p* > 0.05.


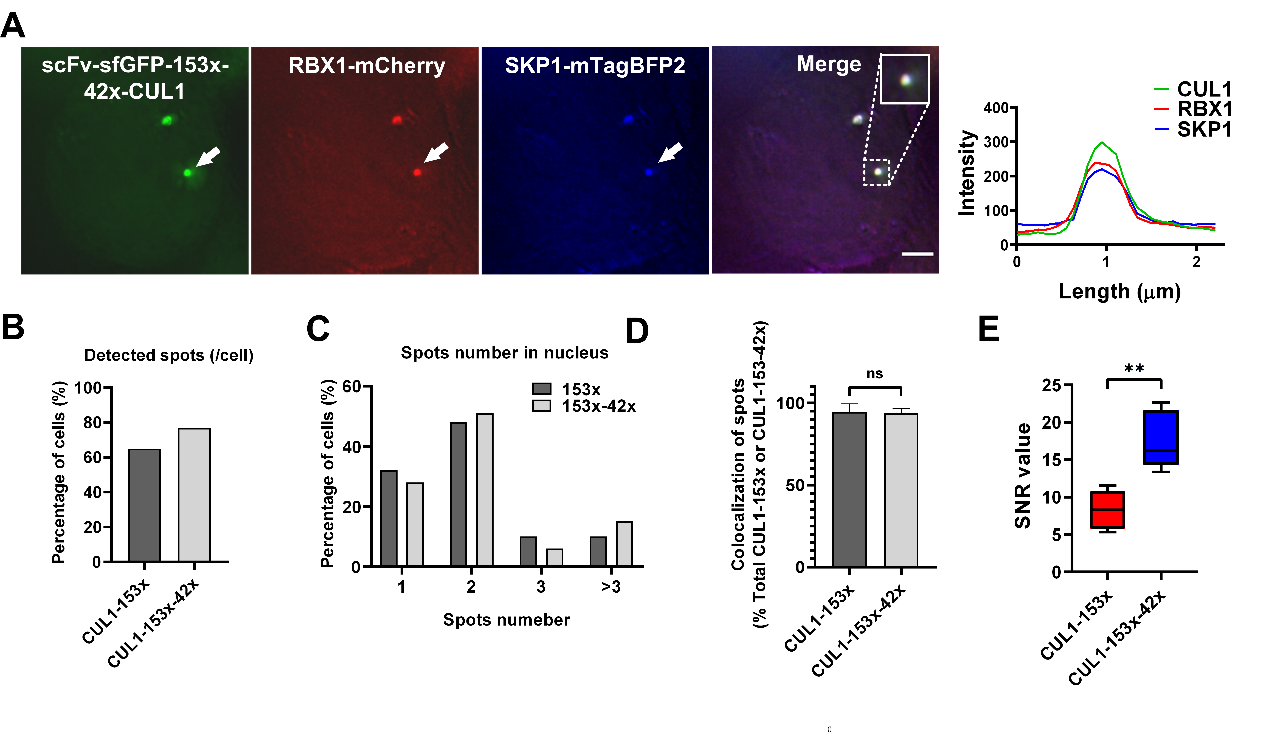


**Supplementary Figure 4. Enhanced brightness of spots by adding 42 repetitive sequences near the target site of *MUC4^-272kb^*.**

**(A)** Representative images depicting the colocalization of 2xNLS-scFv-sfGFP-CUL1 (2xNLS-sfGFP-153x-42x-CUL1, representing the co-transfection of 2xNLS-scFv-sfGFP-CUL1, sgRNA *MUC4^-272kb^*, and sgRNA *MUC4^-272kb-42^*) with RBX1 and SKP1. SunTag10x-dCas9-HEK293T cells were transfected, and images were obtained 24 h later. Dashed boxes show enlarged condensate images, and the right panels correspond to the fluorescence intensity line profiles of the enlarged images. Scale bar, 3 μm.

**(B)** The proportion of cells with successfully formed spots by recruiting 2xNLS-sfGFP-153x-42x-CUL1 and 2xNLS-153x-sfGFP-CUL1, n = 100 cells. Comparison between the two groups was performed using a t-test. “ns” no significance, *p* > 0.05.

**(C)** The distribution percentage of the number of spots of 2xNLS-sfGFP-153x-42x-CUL1, n = 100 cells.

**(D)** Percentage of colocalization of 2xNLS-sfGFP-153x-42x-CUL1 and 2xNLS-153x-sfGFP-CUL1 with RBX1. Graphs show the mean ± SD with three biological replicates and each replicate contains 15 cells.

**(E)** The SNR values of 2xNLS-sfGFP-153x-42x-CUL1 and 2xNLS-sfGFP-153x-CUL1, n = 5 spots. Comparison between the two groups was performed using a t-test. “**” indicates significant difference (*p* < 0.01).


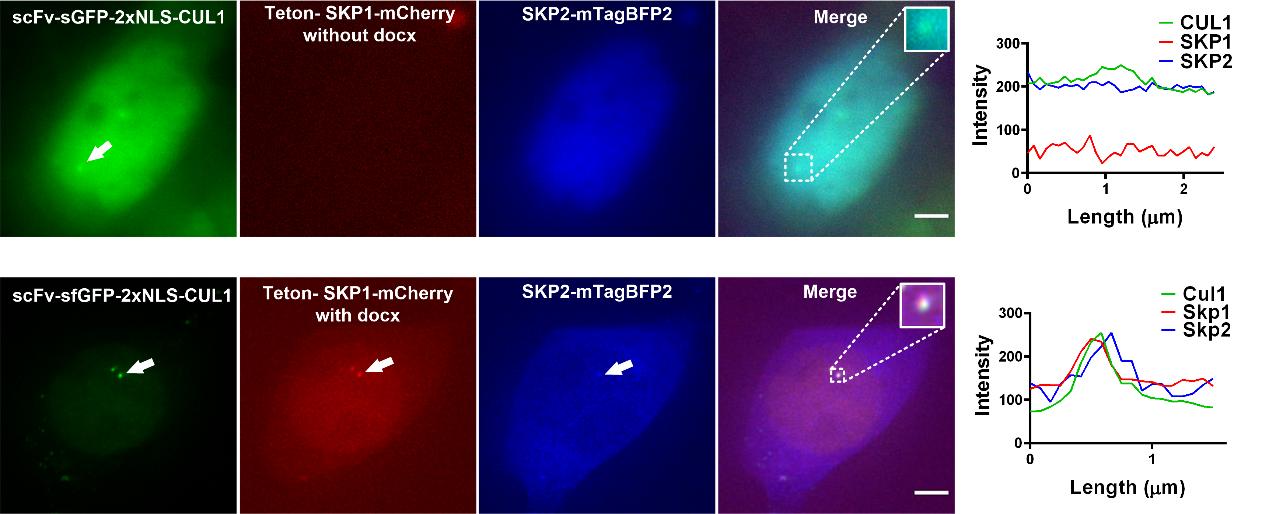


**Supplementary Figure 5. Interactions between CUL1 and SKP2 under the presence or absence of SKP1.** The representative image illustrates the relationship between CUL1 and SKP2 in the presence or absence of SKP1. Only when SKP1 expression is induced by docx, SKP2 colocalizes with CUL1. SunTag10x-dCas9-HEK293T cells were transfected, and images were obtained 24 h later. Dashed boxes show enlarged condensate images, and the right panels correspond to the fluorescence intensity line profiles of the enlarged images. All Scale bars, 3 μm.


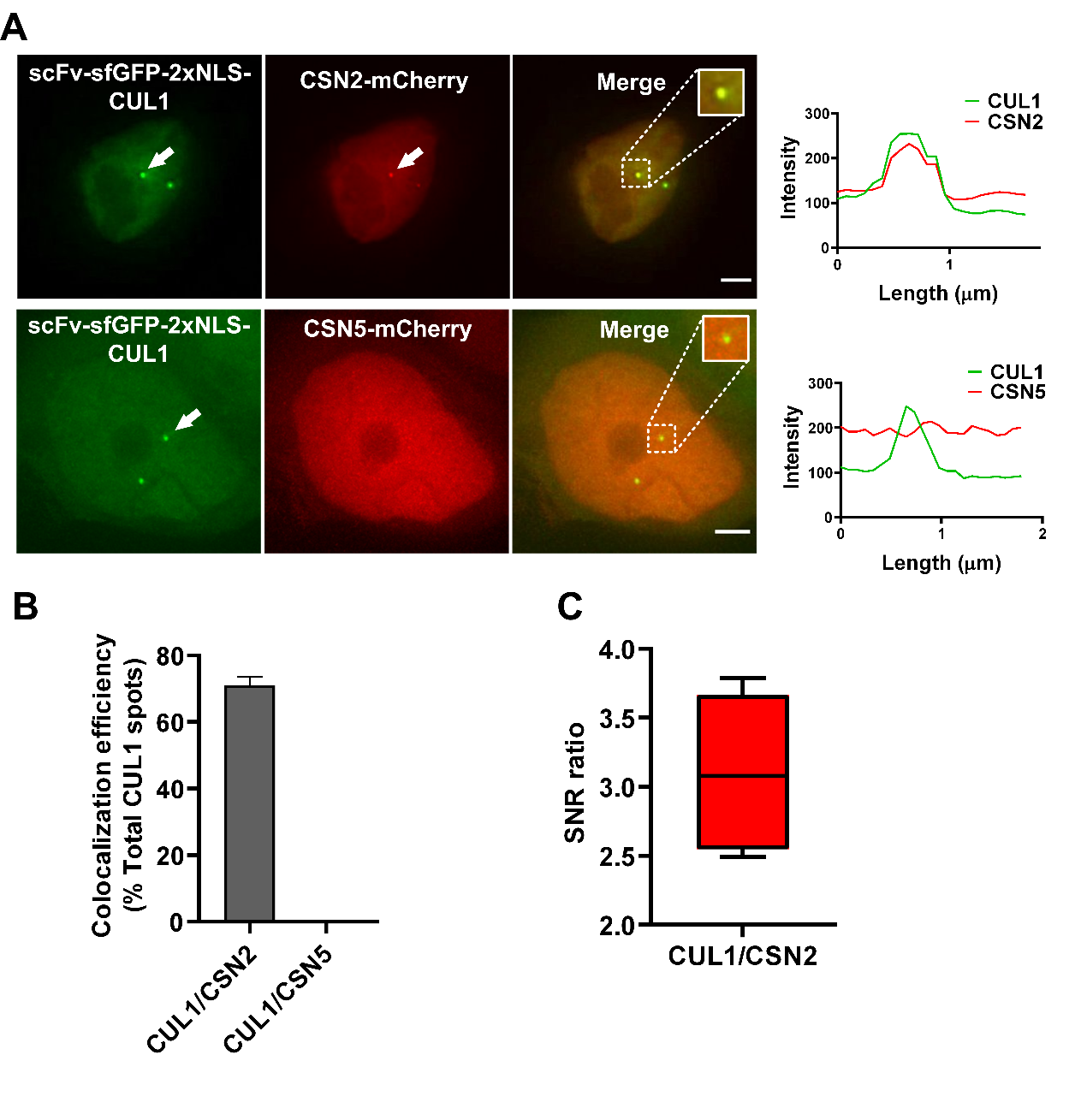


**Supplementary Figure 6. Interactions Between CUL1 and the CSN Complex.**

**(A)** Representative image depicting the interaction between CUL1 and CSN complex components CSN2 and CSN5. CSN2 colocalizes with CUL1, while CSN5 does not colocalize with CUL1. Images were obtained 24 h after SunTag10x-dCas9-HEK293T cells were transfected. Dashed boxes show enlarged condensate images, and the right panels correspond to the fluorescence intensity line profiles of the enlarged images. All Scale bars, 3 μm.

**(B)** Percentage of colocalized spots of 2xNLS-scFv-sfGFP-CUL1 with CSN2 and CSN5. Graphs show the mean ± SD with three biological replicates and each replicate contains 15 cells.

**(C)** The ratio of SNR values between 2xNLS-scFv-sfGFP-CUL1 and CSN2, n = 5 co-localized spots.


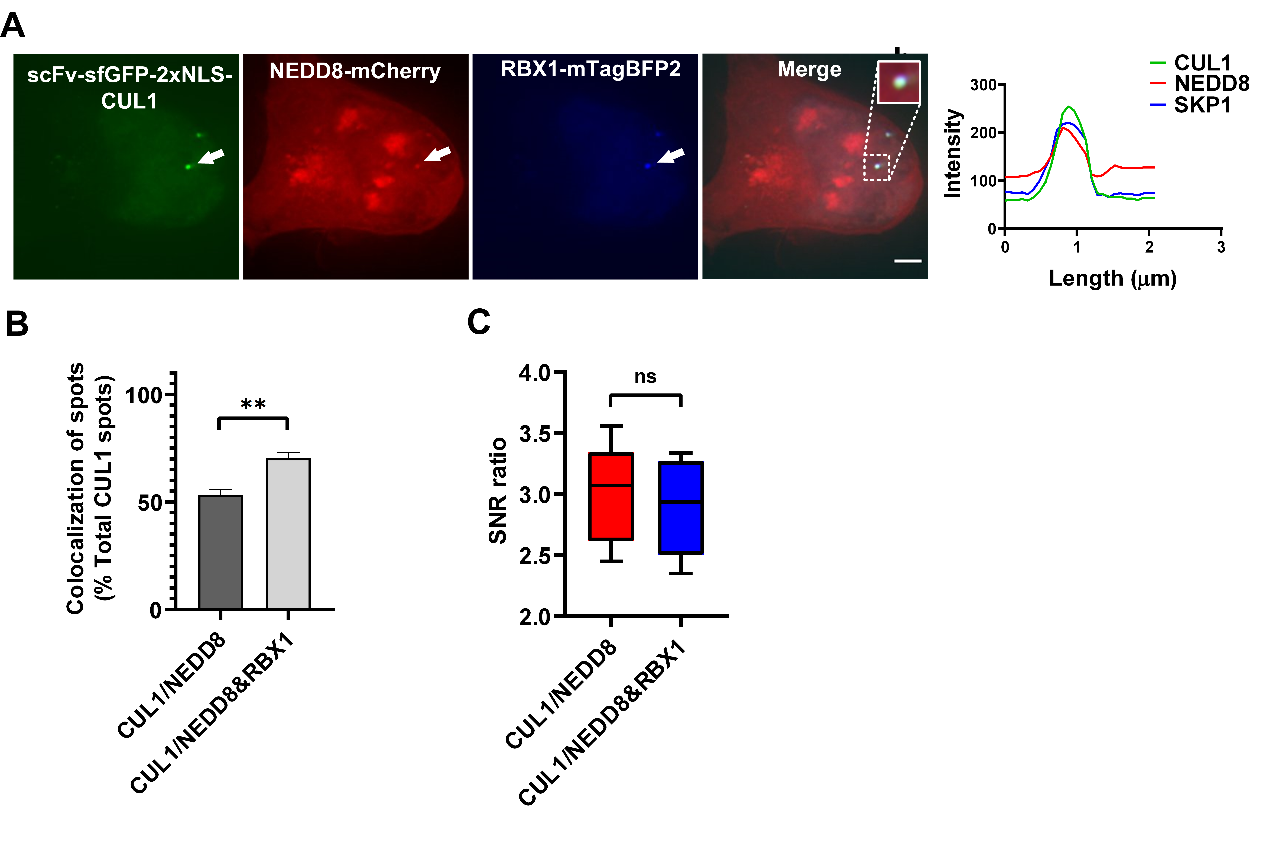


**Supplementary Figure 7. RBX1 was verified by MRC to promote CUL1 binding to NEDD8.**

**(A)** Representative image illustrating the interaction between CUL1, NEDD8, and RBX1. Images were obtained 24 h after SunTag10x-dCas9-HEK293T cells were transfected. Dashed boxes show enlarged condensate images, and the right panels correspond to the fluorescence intensity line profiles of the enlarged images. Scale bar, 3 μm.

**(B)** Percentage of colocalization spots formed. (CUL1/NEDD8 represents the percentage of colocalization between CUL1 and NEDD8 alone, CUL1/NEDD8&RBX1 represents the percentage of colocalization between CUL1 and NEDD8 in the presence of RBX1). The vertical axis represents the average percentage ± SD of colocalization for each protein, with three biological replicates and n = 15 cells per replicate. Comparison between the two groups was performed using a t-test. “**” indicates significant difference (*p* < 0.01).

**(C)** The ratio of SNR values between the co-localized points of CUL1 and NEDD8 alone, as well as in the presence of RBX1, n = 5 co-localized points. Comparison between the two groups was performed using a t-test. “ns” no significance, *p* > 0.05.


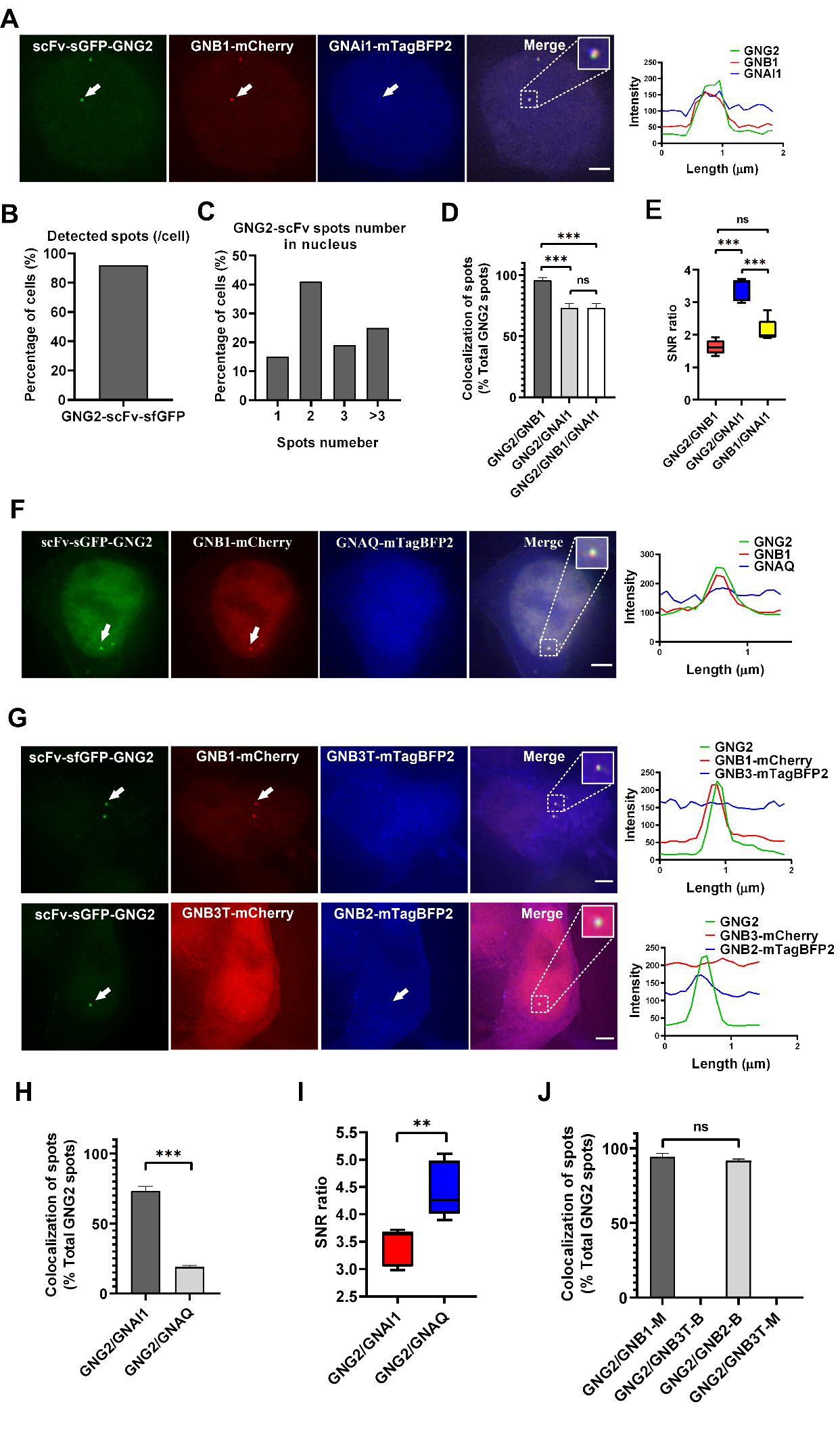


**Supplementary Figure 8. Verification of interactions and visualization of the binding capacity between GNAI and GNAQ with GNB1, and between GNB3T and GNB1 using MRC.**

**(A)-(E)** Verification of interactions among GNAI, GNB1, and GNG2 within the GPCR complex using the MRC.

**(A)** Representative image validates the interactions among G protein subfamily members. Corresponding constructs were co-transfected into SunTag10x-dCas9-HEK293T cells, and images were obtained 24 h later. GNAi colocalized with GNG2 and GNB1. Dashed boxes indicate magnified condensate images, and the right panel shows the fluorescence intensity profiles of the magnified images. Scale bar, 3 μm.

**(B)-(E)** Corresponding to **(A)**.

**(B)** The proportion of cells with formed spots by recruiting GNG2, n = 100 cells

**(C)** The distribution percentage of spots number of GNG2 in cells, n = 100 cells.

**(D)** Percentage of colocalized spots among GNG2, GNGB1, and GNAi. Graphs show the mean ± SD with three biological replicates and each replicate contains 15 cells. Group comparisons were performed using Tukey’s HSD test. “***” indicates significant difference (*p* < 0.001). “ns” no significance, *p* > 0.05.

**(E)** Box plot representing the ratio of SNR values among GNG2, GNGB1, and GNAi (n = 5 colocalization points). Group comparisons were performed using Tukey’s HSD test. “***” indicates significant difference (*p* < 0.001). “ns” no significance, *p* > 0.05.

**(F), (H) and (I)** Verification of interactions among GNAQ, GNB1, and GNG2 within the GPCR complex using the MRC, and comparison of colocalization and SNR ratios between GNAI and GNAQ.

**(G) and (J)** Verification of interactions among GNB3T, GNB1, and GNG2 within the GPCR complex using the MRC

**(F)** Representative image shows GNAQ doesn’t co-localize with GNG2 and GNB1 spots. The majority of cells did not form colocalized points of GNAQ with GNG2 and GNB1. SunTag10x-dCas9-HEK293T cells were transfected with corresponding constructs, and images were obtained 24 h later.

**(G)** Representative image shows that GN3T does not colocalize with GNG2 and GNB1 spots. SunTag10x-dCas9-HEK293T cells were transfected, and images were obtained 24 h later. All Scale bars, 3 μm.

**(H) and (I)** corresponding to **(F)**. **(H),** Percentage of colocalization between GNG2 with GNAI1 and GNAQ. Graphs show the mean ± SD with three biological replicates and each replicate contains 15 cells. Comparison between the two groups was performed using a t-test. “***” indicates significant difference (*p* < 0.001).

**(I)** Box plots represent the ratio of SNR values between GNG2 and GNAI1, GNAQ interactions, n = 5 colocalized points. Comparison between the two groups was performed using a t-test. “**” indicates significant difference (*p* < 0.01).

**(J)** corresponding to **(G)**, average percentage ± SD of colocalization between GNG2 and GNB2, GNB3T, as shown in the figure, with three biological replicates and n = 15 cells per replicate. Comparison between the two groups was performed using a t-test. “ns” no significance, *p* > 0.05.


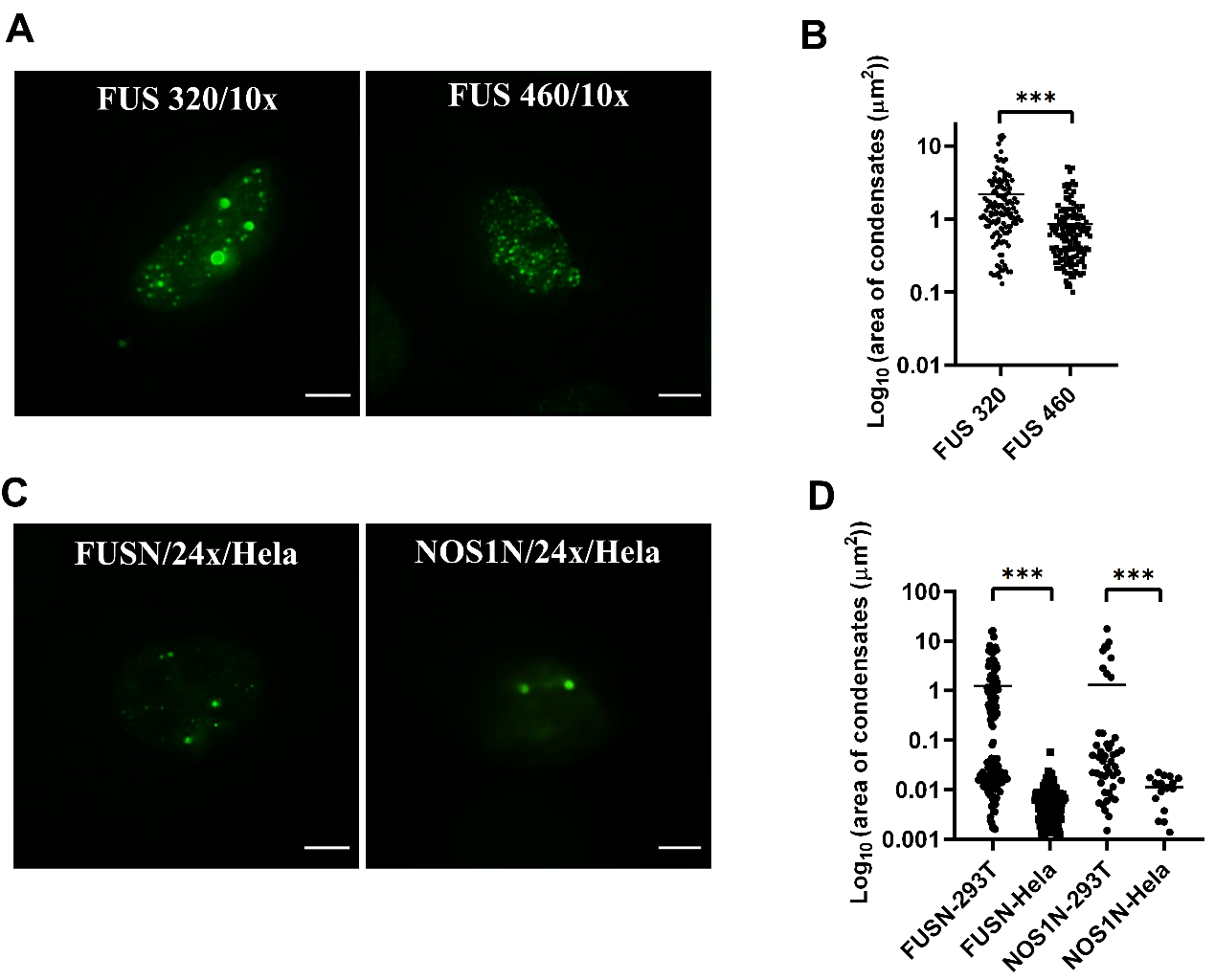


**Supplementary Figure 9. Formation of condensates by different fragments length of FUSN.**

**(A)** Live-cell imaging of condensates formed by various lengths of FUSN fragments. SunTag10x-dCas9-HEK293T cells were transfected with specific FUSN fragments, and images were obtained 24 h later. All scale bars, 5μm.

**(B)** Quantification of the condensate area formed by FUSN 320 and FUSN 460. The scatter plot represents the log10 mean value of the formed condensate area. Each point represents a condensate (n = 5 cells).

**(C)** Live-cell imaging of condensates formed by FUSN and NOS1N in HeLa cells. The scale bar is 5 μm. Specific FUSN or NOS1N was co-transfected with SunTag24x-dCas9 in HeLa cells, and images were obtained 24 h later. Comparison between the two groups was performed using a t-test .“**” indicates significant difference (*p* < 0.01).

**(D)** Scatter plot representing the log10 mean value of the condensate area formed by FUSN and NOS1N in both HeLa and HEK293T cells. Each point represents a condensate (n = 5 cells). Comparison between the two groups was performed using a t-test. “**” indicates significant difference (*p* < 0.01).


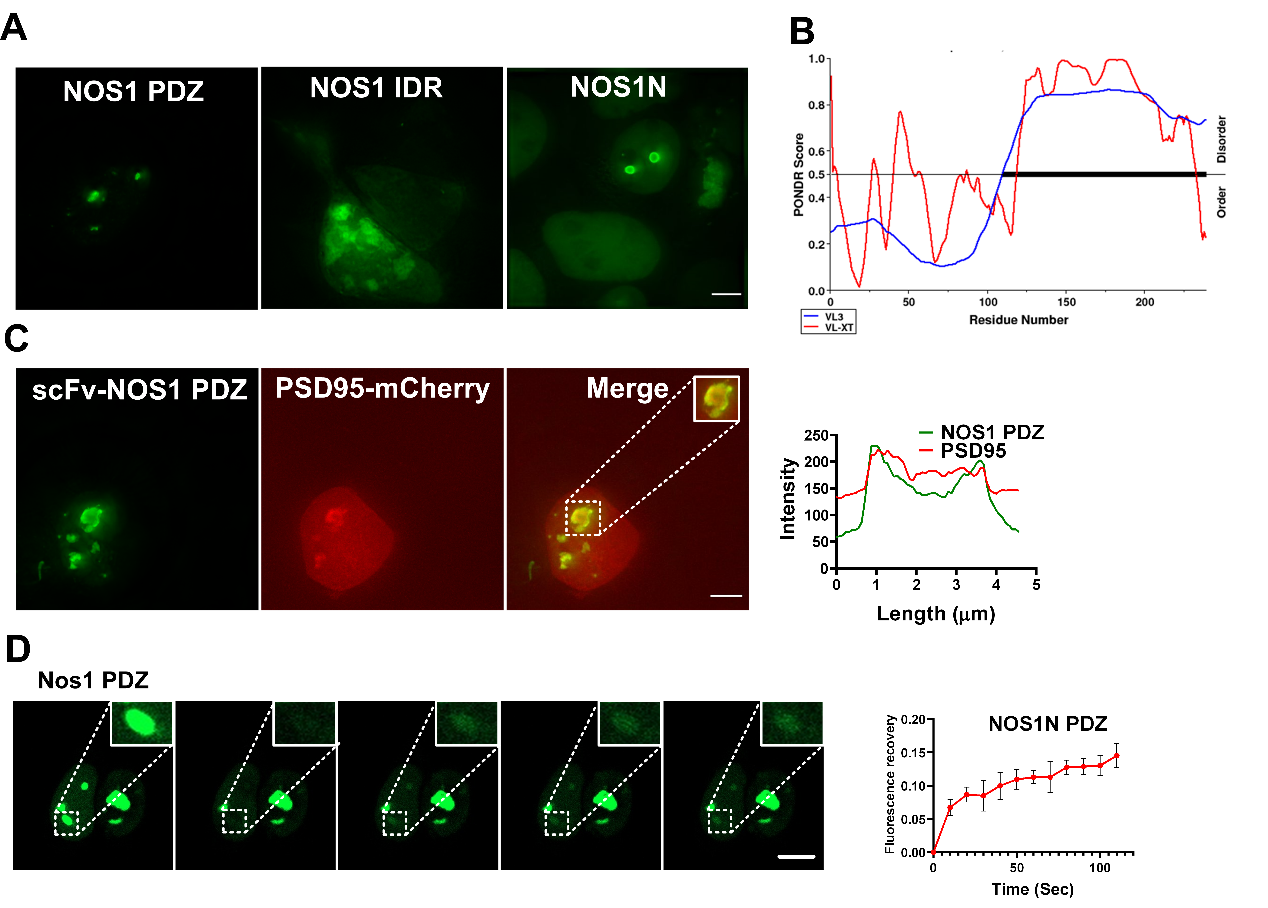


**Supplementary Figure 10. Validation the formation of phase-separated condensates by PDZ domain and IDR of NOS1N using MRC.**

**(A)** Representative live-cell image of NOS1 PDZ, NOS1 IDR, and NOS1N in the SunTag24x system. Scale bar, 5 μm.

**(B)** Disordered score assessment of different regions of NOS1N. An IDR segment from amino acid position 100 to 235 was predicted by PONDR.

**(C)** Representative live-cell imaging of NOS1 PDZ co-transfected with PSD95 in SunTag24x-dCas9-HEK293T cells. Images were captured 24 h after transfection using the same imaging settings. Dashed boxes show enlarged condensate images, and the right panels correspond to the fluorescence intensity line profiles of the enlarged images. All Scale bars, 5 μm.

**(D)** FRAP analysis of NOS1 PDZ in cells. Dashed squares indicate the photobleached region. The scale bar is 5 μm. The right panel represents the quantification of fluorescence measurements of NOS1 PDZ condensates' contraction over time after photobleaching. Background-subtracted fluorescence measurements are plotted. Values represent mean ± SD (n = 5).


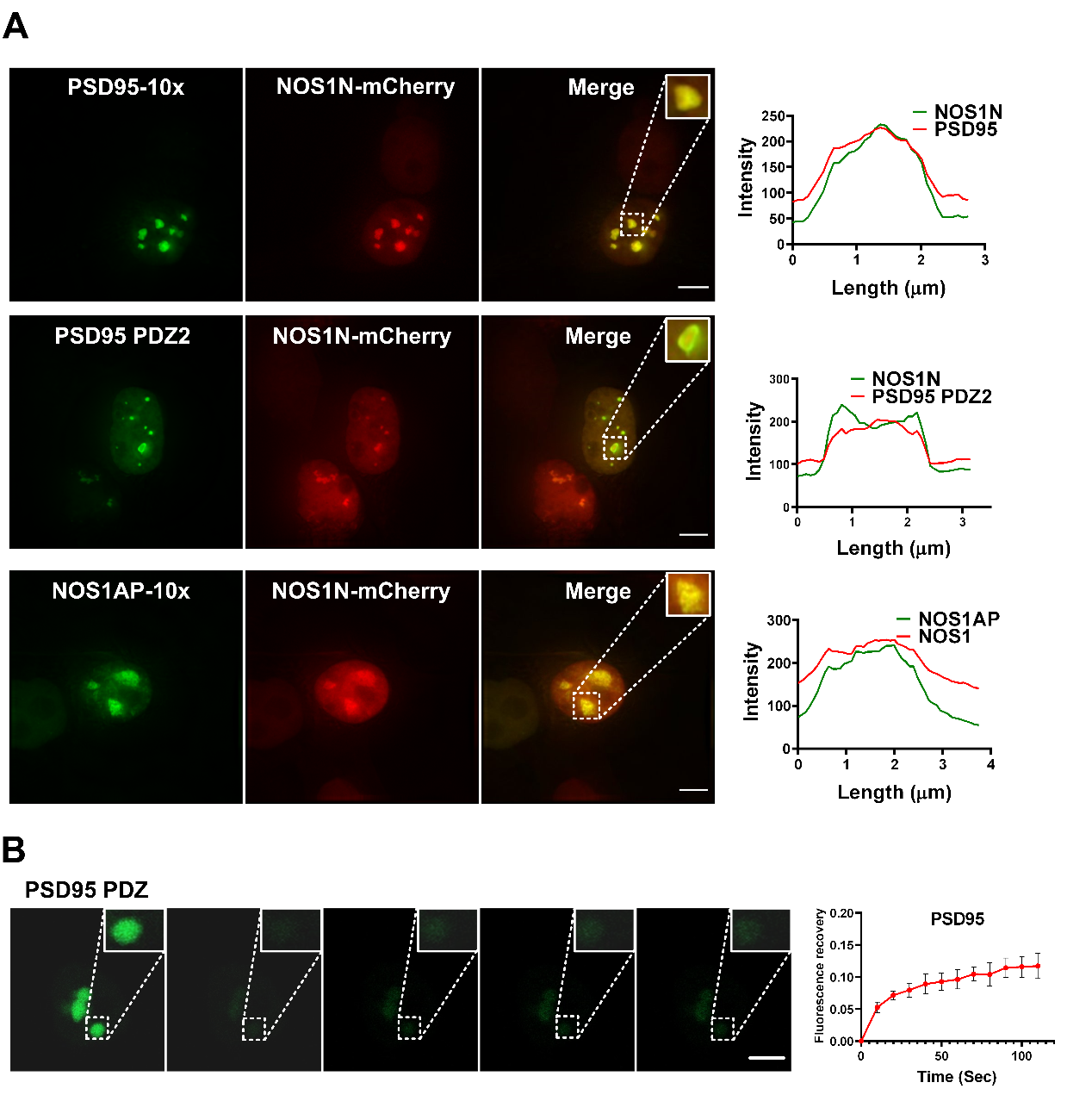


**Supplementary Figure 11. Interactions of PSD95, PSD95 PDZ2, NOS1AP, and NOS1N-mCherry in SunTag10x system.**

**(A)** Representative images captured 24 h after co-transfection of PSD95, PSD95 PDZ2, NOS1AP, and NOS1N-mCherry in SunTag10x-dCas9-HEK293T cells. Colocalization of PSD95, PSD95 PDZ2, NOS1AP, and NOS1N-mCherry is observed. Dashed boxes show enlarged condensate images, and the right panels correspond to the fluorescence intensity line profiles of the enlarged images. All Scale bars, 5 μm.

**(B)** FRAP analysis of PSD95 PDZ2 in cells. Dashed squares indicate the photobleached region. The scale bar is 5 μm. The right panel represents the quantification of fluorescence measurements of PSD95 PDZ2 condensates' contraction over time after photobleaching. Background-subtracted fluorescence measurements are plotted. Values represent mean ± SD (n = 5).


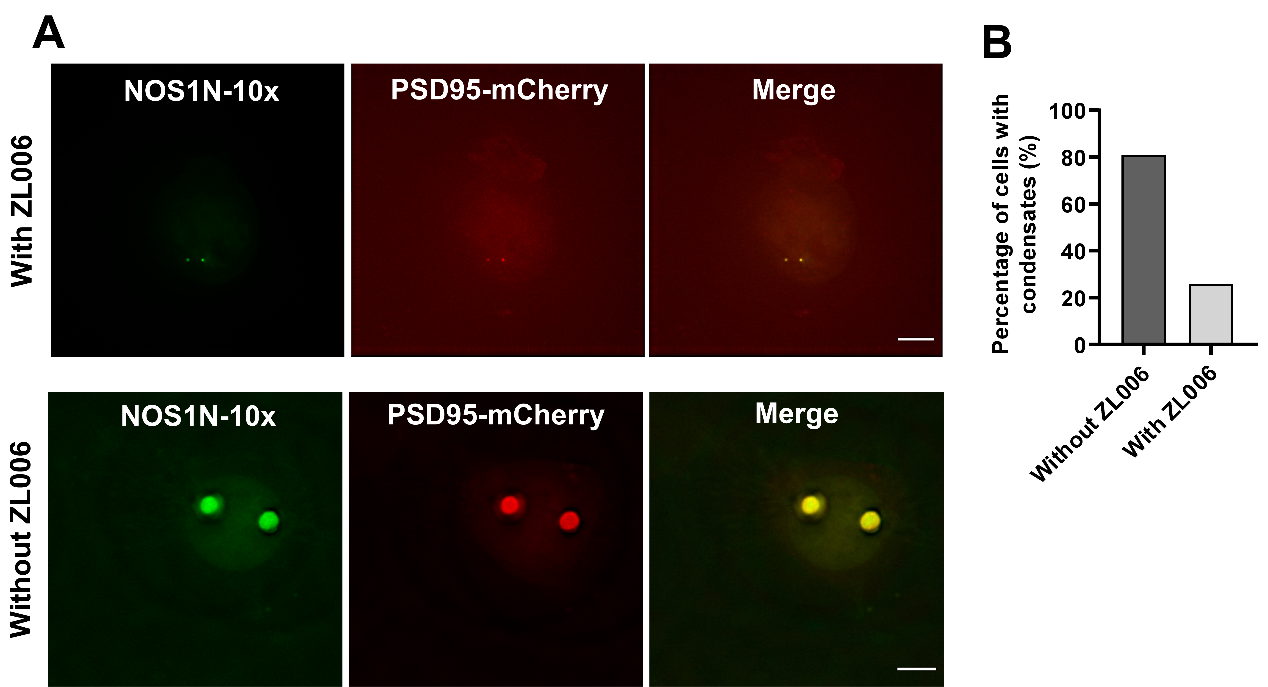


**Supplementary Figure 12. The effect of ZL006 on the formation of condensates in NOS1N-PSD95 complex.**

**(A)** Representative images illustrating the impact of adding ZL006 on the formation of condensates in the NOS1N-PSD95 complex. NOS1N and PSD95 were co-transfected into SunTag10x-dCas9-HEK293T cell, and imaging was performed with or without the addition of ZL006 after 24 h. All Scale bars, 3μm.

**(B)** corresponding to (A). The percentage of cells with NOS1N condensates in the presence or absence of ZL006 (n = 100).


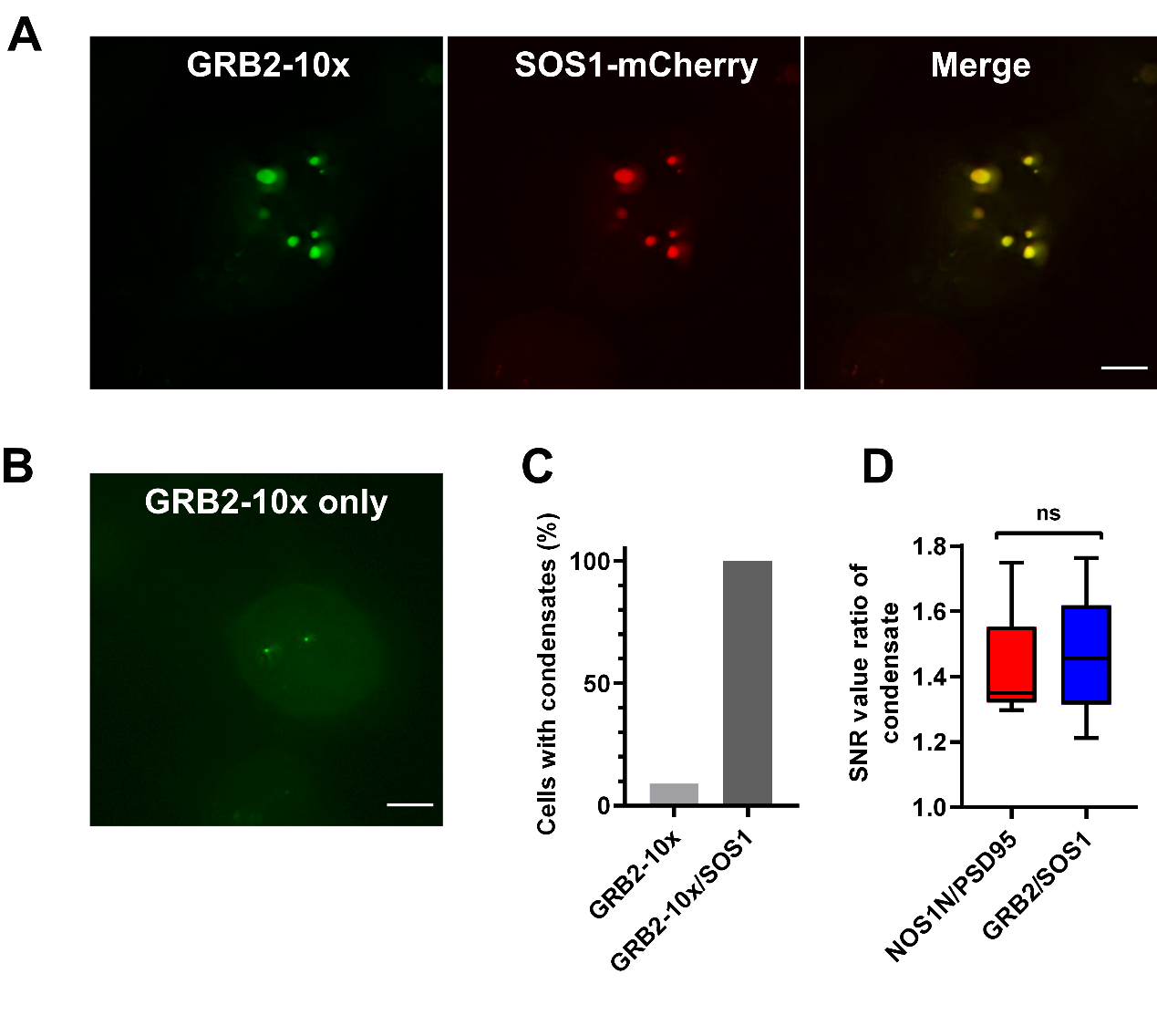


**Supplementary Figure 13.** Representative images of co-transfection of GRB2 with SOS1 **(A)** and single transfection of GRB2 **(B)** in SunTag10x-dCas9-HEK293T cells. The images were obtained using the same imaging settings after transfection. All scale bars, 5μm.

**(C)** Percentage of cells with condensates formed after co-transfection of GRB2 with SOS1or single transfection of GRB2 in SunTag10x-dCas9-HEK293T cells (n = 100 cells).

**(D)** Box plot showing the SNR ratio within co-localized condensates for NOS1N/PSD95 and GRB2/SOS1. For NOS1N/PSD95, the SNR of NOS1N was divided by that of PSD95; for GRB2/SOS1, the SNR of GRB2 was divided by that of SOS1. *n* = 5 co-localized condensates. Comparison between the two groups was performed using a t-test. “ns” no significance, *p* > 0.05.


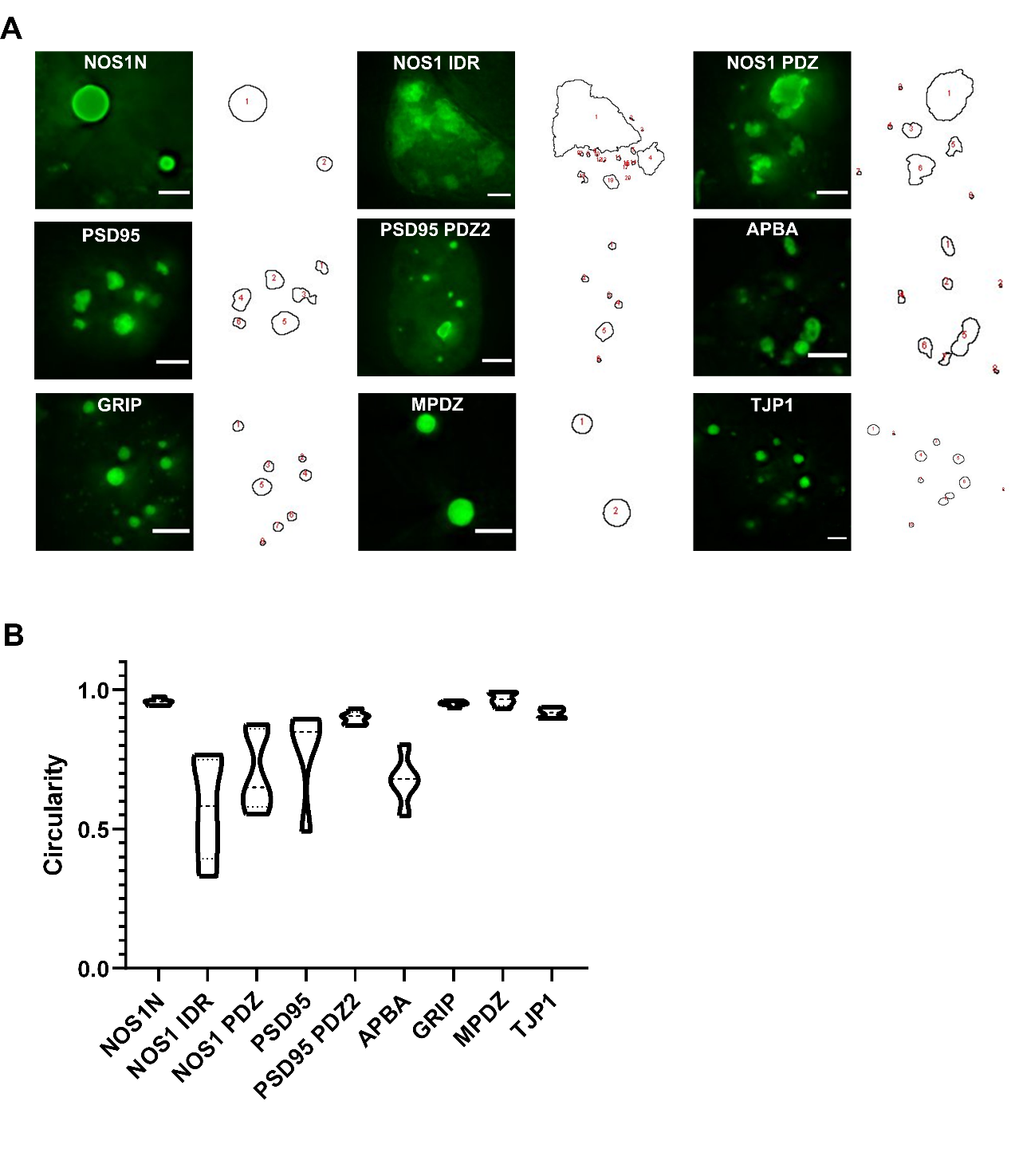


**Supplementary Figure 14. Analysis of the circularity of protein condensates.**

**(A)** Representative images showing the morphology of condensates formed by different proteins. The boundaries of individual condensates were identified and outlined using ImageJ. All scale bars, 3 μm.

**(B)** Violin plots represent the circularity values of condensates formed by each protein, n = 5 condensates. Circularity was quantified using ImageJ based on the identified shapes.


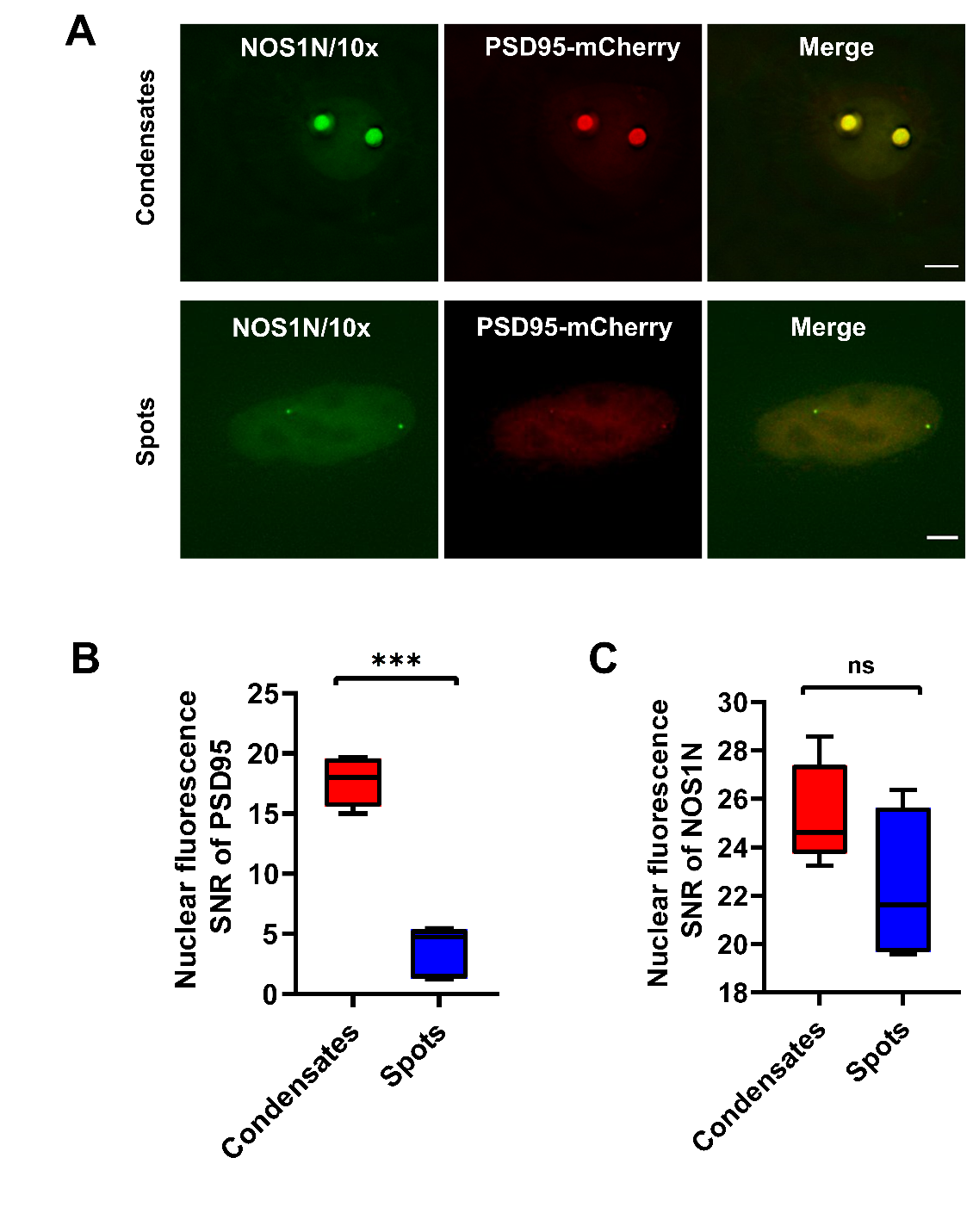


**Supplementary Figure 15. Comparison of nuclear fluorescence between NOS1N–PSD95 condensate-forming and non-condensate cells.**

**(A)** Representative images showing two distinct outcomes upon co-expression of NOS1N and PSD95: formation of condensates (droplet-like structures) and non-condensate spots.

Box plot comparing nuclear fluorescence SNR of PSD95 **(B)** and NOS1N **(C)** between cells forming condensates and those displaying only puncta upon co-expression of NOS1N and PSD95, n = 5 colocalized condensates or spot. Comparison between the two groups was performed using a t-test. “*” *p* < 0.05. Higher SNR is observed in condensate-forming cells, suggesting enhanced recruitment and concentration of fluorescent signal. “ns” no significance, *p* > 0.05.


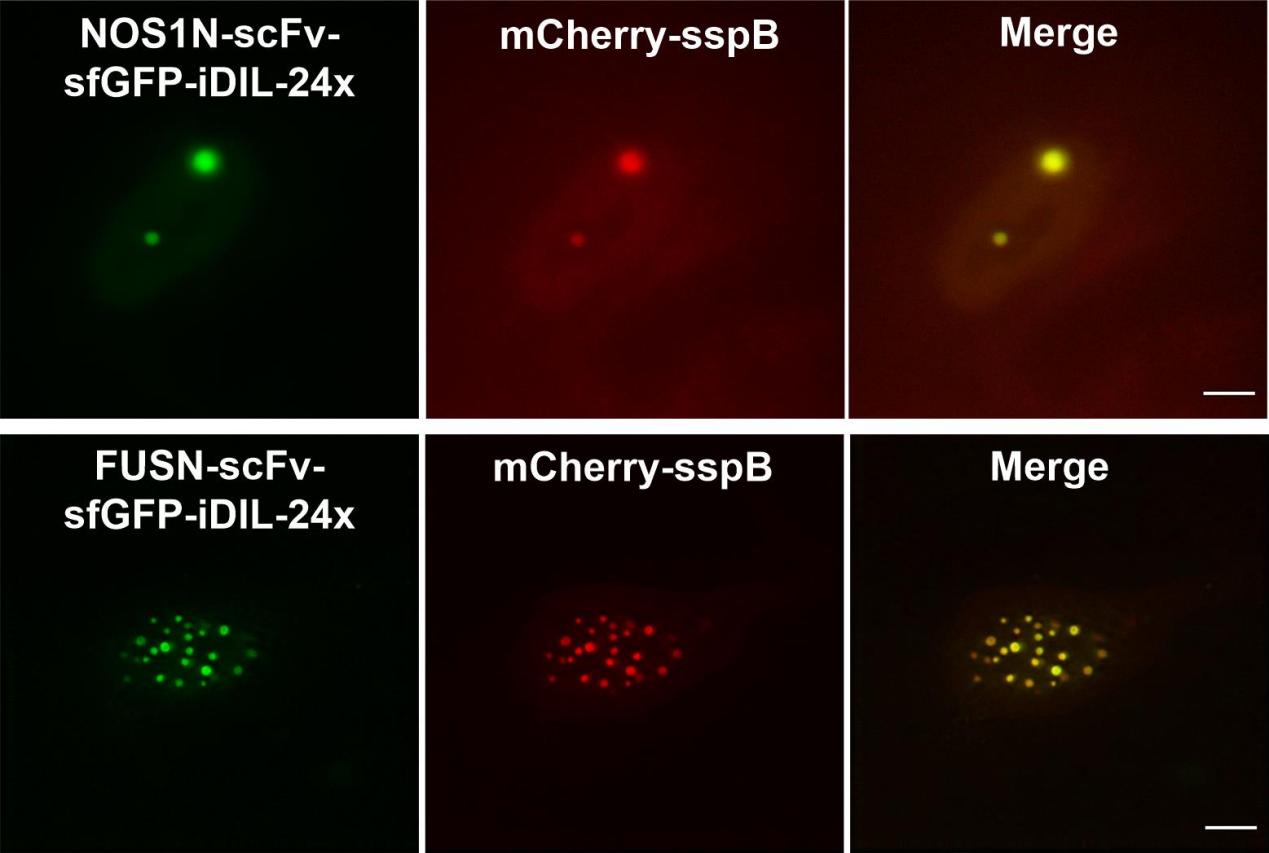


**Supplementary Figure 16. MRC combined with optogenetic tools enables creating different condensates within cells and introducing exogenous proteins into condensates.** NOS1N-scFv-sfGFP-iLID-24x and FUS1N-scFv-sfGFP-iLID-24x represent fusion constructs of NOS1N-scFv-sfGFP and FUS1N-scFv-sfGFP with iLID. These constructs induce the formation of green condensates within the SunTag24x system. Upon interaction between the optogenetic tools iLID and sspB, mCherry-sspB is recruited into the condensates. All experiments were conducted in the SunTag24x-dCas9-HEK293T cell. All Scale bars, 5μm.


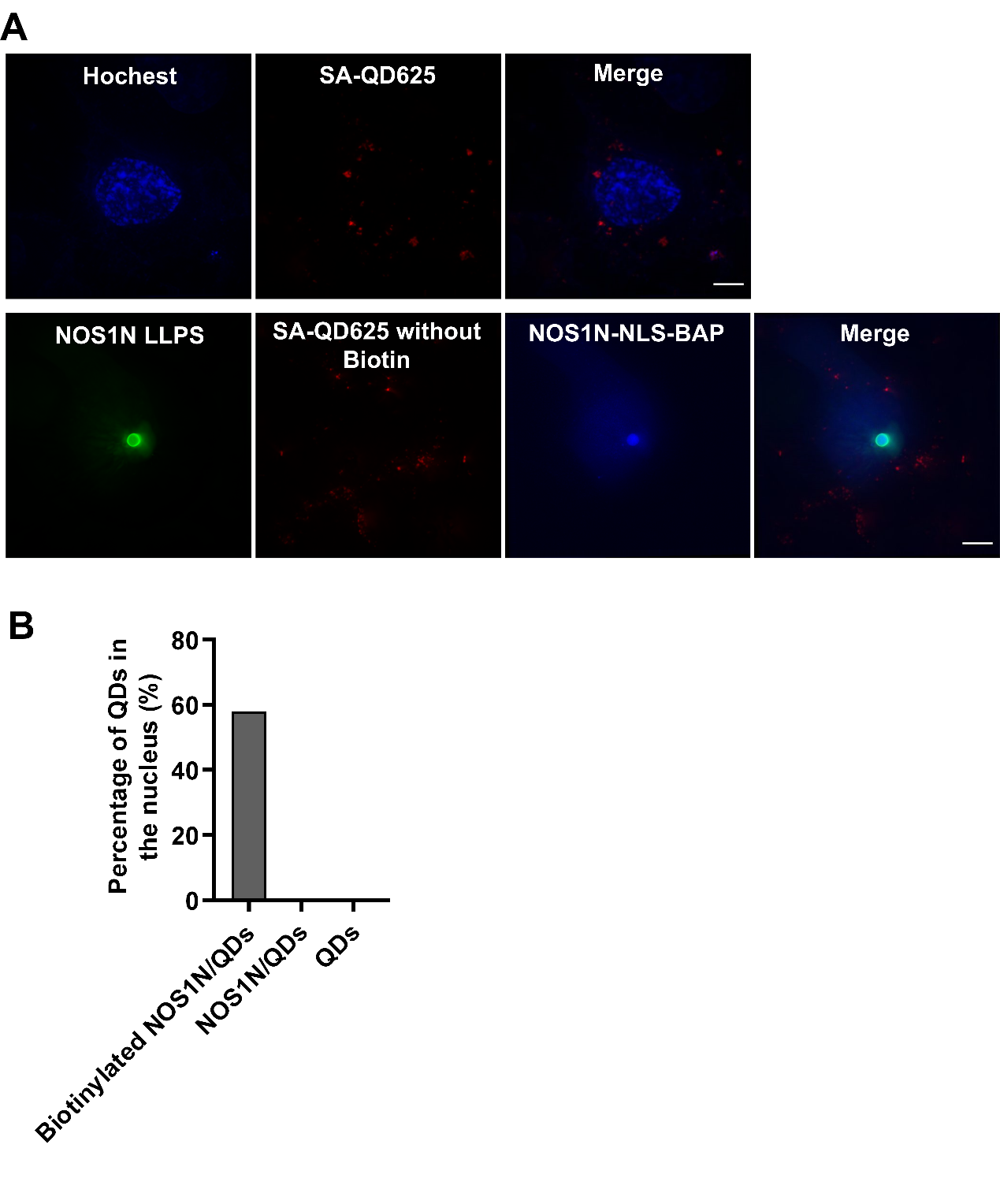


**Supplementary Figure 17.** **SA-QD605 transfection alone NOS1N-NLS-BAP without biotinylated do not lead to nuclear entry of QDs.** **(A)** Representative images of cells transfected with the indicated constructs. Nuclei were stained with Hoechst dye. NOS1N forms condensates. Co-transfected NOS1N-NLS-BAP and SA-QD605 without biotin supplementation do not interact, and QDs cannot enter the nucleus. All experiments were conducted in the SunTag24x-dCas9-HEK293T cell. All scale bars are 5 μm. **(B)** Percentage of QDs in the nucleus under different conditions. Biotinylated NOS1N/QDs: Biotinylated NOS1N-NLS-BAP combined with SA-QD605, NOS1N/QDs: the combination of non-biotinylated NOS1N-NLS-BAP and QDs, QDs: single transfection of SA-QD605.

**Supplementary Table 1. The number of spots detected for the respective proteins and their colocalized spots.** The table provides information on two proteins (Protein 1 and Protein 2) and their respective spot counts, including the individual spot counts for each protein (Protein 1 spots count and Protein 2 spots count), as well as the count of colocalized spots (Colocalized Count), n = 15 cells.

| Protein 1 | Protein 2 | Protein 1 spots count | Protein 2 spots count | Colocalized Count |
| --- | --- | --- | --- | --- |
| scFv-sfGFP-SpyCatcher | SpyTag-mCherry | 27 | 26 | 26 |
| scFv-sfGFP-SpyCatcher | SpyTag-mCherry | 28 | 25 | 25 |
| scFv-sfGFP-SpyCatcher | SpyTag-mCherry | 25 | 23 | 23 |
| scFv-sfGFP-bJun | bFos-mCherry | 39 | 42 | 34 |
| scFv-sfGFP-bJun | bFos-mCherry | 37 | 40 | 34 |
| scFv-sfGFP-bJun | bFos-mCherry | 41 | 45 | 37 |
| scFv-sGFP-2xNLS-Cul1 | RBX1-mCherry | 29 | 29 | 28 |
| scFv-sGFP-2xNLS-Cul1 | RBX1-mCherry | 32 | 29 | 29 |
| scFv-sGFP-2xNLS-Cul1 | RBX1-mCherry | 30 | 27 | 27 |
| scFv-sGFP-2xNLS-Cul1 | SKP1-mTagBFP2 | 29 | 23 | 23 |
| scFv-sGFP-2xNLS-Cul1 | SKP1-mTagBFP2 | 32 | 26 | 26 |
| scFv-sGFP-2xNLS-Cul1 | SKP1-mTagBFP2 | 30 | 25 | 25 |
| scFv-sfGFP-GNG2 | GNB1-mCherry | 29 | 27 | 27 |
| scFv-sfGFP-GNG2 | GNB1-mCherry | 33 | 31 | 31 |
| scFv-sfGFP-GNG2 | GNB1-mCherry | 30 | 28 | 28 |
| scFv-sfGFP-GNG2 | GNB2-mTagBFP2 | 29 | 25 | 25 |
| scFv-sfGFP-GNG2 | GNB2-mTagBFP2 | 33 | 28 | 28 |
| scFv-sfGFP-GNG2 | GNB2-mTagBFP2 | 30 | 26 | 26 |
| scFv-mCherry-SKP1 | CUL1-GN with RBX1-GC | 27 | 20 | 20 |
| scFv-mCherry-SKP1 | CUL1-GN with RBX1-GC | 26 | 18 | 18 |
| scFv-mCherry-SKP1 | CUL1-GN with RBX1-GC | 28 | 18 | 18 |
| scFv-mCherry-SKP1 | SKP2-mTagBFP | 27 | 16 | 16 |
| scFv-mCherry-SKP1 | SKP2-mTagBFP | 26 | 15 | 15 |
| scFv-mCherry-SKP1 | SKP2-mTagBFP | 28 | 15 | 15 |
| scFv-sfGFP-SKP1 | RBX1-mCherry | 26 | 0 | 0 |
| scFv-sfGFP-SKP1 | RBX1-mCherry | 26 | 0 | 0 |
| scFv-sfGFP-SKP1 | RBX1-mCherry | 27 | 0 | 0 |
| scFv-sfGFP-GNG2 | GNB2-mCherry | 28 | 26 | 26 |
| scFv-sfGFP-GNG2 | GNB2-mCherry | 30 | 27 | 27 |
| scFv-sfGFP-GNG2 | GNB2-mCherry | 28 | 24 | 24 |
| scFv-sfGFP-GNG2 | GNB1-mTagBFP2 | 28 | 23 | 23 |
| scFv-sfGFP-GNG2 | GNB1-mTagBFP2 | 30 | 26 | 26 |
| scFv-sfGFP-GNG2 | GNB1-mTagBFP2 | 28 | 24 | 24 |
| scFv-sfGFP-CUL1 | RBX1-mCherry | 9 | 8 | 8 |
| scFv-sfGFP-CUL1 | RBX1-mCherry | 8 | 7 | 7 |
| scFv-sfGFP-CUL1 | RBX1-mCherry | 10 | 8 | 8 |
| scFv-sfGFP-CUL1-2xGCN | RBX1-mCherry | 5 | 3 | 3 |
| scFv-sfGFP-CUL1-2xGCN | RBX1-mCherry | 5 | 3 | 3 |
| scFv-sfGFP-CUL1-2xGCN | RBX1-mCherry | 4 | 2 | 2 |
| scFv-sfGFP-CUL1-24xGCN | RBX1-mCherry | 36 | 33 | 33 |
| scFv-sfGFP-CUL1-24xGCN | RBX1-mCherry | 38 | 36 | 36 |
| scFv-sfGFP-CUL1-24xGCN | RBX1-mCherry | 32 | 33 | 31 |
| scFv-sfGFP-153x-42x-CUL1 | RBX1-mCherry | 26 | 24 | 24 |
| scFv-sfGFP-153x-42x-CUL1 | RBX1-mCherry | 30 | 29 | 29 |
| scFv-sfGFP-153x-42x-CUL1 | RBX1-mCherry | 28 | 26 | 26 |
| scFv-sfGFP-2xNLS-CUL1 | CSN2-mCherry | 26 | 19 | 19 |
| scFv-sfGFP-2xNLS-CUL1 | CSN2-mCherry | 28 | 19 | 19 |
| scFv-sfGFP-2xNLS-CUL1 | CSN2-mCherry | 28 | 15 | 15 |
| scFv-sfGFP-2xNLS-CUL1 | CSN5-mCherry | 29 | 0 | 0 |
| scFv-sfGFP-2xNLS-CUL1 | CSN5-mCherry | 26 | 0 | 0 |
| scFv-sfGFP-2xNLS-CUL1 | CSN5-mCherry | 28 | 0 | 0 |
| scFv-sfGFP-GNG2 | GNAi1-mTagBFP2 | 28 | 20 | 20 |
| scFv-sfGFP-GNG2 | GNAi1-mTagBFP2 | 35 | 27 | 27 |
| scFv-sfGFP-GNG2 | GNAi1-mTagBFP2 | 29 | 21 | 21 |
| scFv-sfGFP-GNG2 | GNAQ-mTagBFP2 | 27 | 6 | 6 |
| scFv-sfGFP-GNG2 | GNAQ-mTagBFP2 | 30 | 8 | 8 |
| scFv-sfGFP-GNG2 | GNAQ-mTagBFP2 | 28 | 7 | 7 |
| scFv-sfGFP-GNG2 | GNB3T-TagBFP2 | 28 | 0 | 0 |
| scFv-sfGFP-GNG2 | GNB3T-TagBFP2 | 31 | 0 | 0 |
| scFv-sfGFP-GNG2 | GNB3T-TagBFP2 | 29 | 0 | 0 |
| scFv-sfGFP-2xNLS-CUL1 | NEDD8-mCherry | 25 | 14 | 14 |
| scFv-sfGFP-2xNLS-CUL1 | NEDD8-mCherry | 29 | 15 | 15 |
| scFv-sfGFP-2xNLS-CUL1 | NEDD8-mCherry | 27 | 13 | 13 |
| scFv-sfGFP-2xNLS-CUL1 | NEDD8-mCherry&RBX1-mTagBFP2 | 27 | 19 | 19 |
| scFv-sfGFP-2xNLS-CUL1 | NEDD8-mCherry&RBX1-mTagBFP2 | 28 | 19 | 19 |
| scFv-sfGFP-2xNLS-CUL1 | NEDD8-mCherry&RBX1-mTagBFP2 | 26 | 17 | 17 |

**Supplementary Table 2.** The table summarizes the percentage of colocalized spots for various protein pairs or complexes, expressed as mean ± standard deviation (SD), n = 15 cells.

| Protein | Colocalized Spots (%), Mean ± SD |
| --- | --- |
| scFv-sfGFP-SpyCatcher/SpyTag-mCherry | 93.0 ± 3.00 |
| scFv-sfGFP-bJun/bFos-mCherry | 89.33 ±2.08 |
| scFv-sGFP-2xNLS-Cul1/RBX1-mCherry | 94.67 ± 4.73 |
| scFv-sGFP-2xNLS-Cul1/SKP1-mTagBFP2 | 81.33 ± 1.53 |
| scFv-sGFP-2xNLS-Cul1/RBX1-mCherry/SKP1-mTagBFP2 | 81.33 ± 1.53 |
| scFv-sfGFP-GNG2/GNB1-mCherry | 93.00 ± 1.00 |
| scFv-sfGFP-GNG2/GNB2-mTagBFP2 | 86.67 ± 1.53 |
| scFv-sfGFP-GNG2/GNB1-mTagBFP2 | 89.33 ± 4.04 |
| scFv-sfGFP-GNG2/GNB2-mCherry | 84.67 ± 2.52 |
| scFv-mCherry-SKP1/CUL1-GN with RBX1-GC | 70.33 ± 3.22 |
| scFv-mCherry-SKP1/SKP2-mTagBFP2 | 60.00 ± 2.65 |
| scFv-mCherry-SKP1/CUL1-GN with RBX1-GC/SKP2-mTagBFP2 | 60.00 ± 2.65 |
| scFv-sfGFP-1xNLS-CUL1/RBX1-mCherry | 84.67 ± 1.53 |
| scFv-sfGFP-CUL1-2xGCN/RBX1-mCherry | 51.67 ± 7.64 |
| scFv-sfGFP-CUL1-10xGCN/RBX1-mCherry | 94.67 ± 4.73 |
| scFv-sfGFP-CUL1-24xGCN/RBX1-mCherry | 94.67 ± 2.52 |
| scFv-sfGFP-CUL1-153xRepeat/RBX1-mCherry | 94.67 ± 4.73 |
| scFv-sfGFP-CUL1-153x-42xRepeat/RBX1-mCherry | 94.00 ± 2.65 |
| scFv-sfGFP-2xNLS-CUL1/CSN2-mCherry | 71.00 ± 2.65 |
| scFv-sGFP-GNG2/GNB1-mCherry | 95.67 ± 2.31 |
| scFv-sGFP-GNG2/GNAi1-mTagBFP2 | 73.33 ± 3.22 |
| scFv-sGFP-GNG2/GNB1-mCherry/GNAi1-mTagBFP2 | 73.33 ± 3.22 |
| scFv-sGFP-GNG2/GNB1-mCherry | 94.33 ± 2.31 |
| scFv-sGFP-GNG2/GNB2-mTagBFP2 | 91.67 ± 1.16 |
| scFv-sfGFP-2xNLS-CUL1/NEDD8-mCherry | 53.33 ± 2.52 |
| scFv-sfGFP-2xNLS-CUL1/NEDD8- mCherry&RBX1- mTagBFP2 | 70.33 ± 2.52 |

**Supplementary Table 3.** The table summarizes the SNR value ratios for various protein pairs, expressed as mean ± standard deviation (SD), n = 5 colocalized spots.

| Protein | SNR value ratio, Mean ± SD |
| --- | --- |
| scFv-sfGFP-SpyCatcher/SpyTag-mCherry | 2.39 ± 0.55 |
| scFv-sfGFP-bJun/bFos-mCherry | 2.94 ± 1.01 |
| scFv-sGFP-2xNLS-Cul1/RBX1-mCherry | 1.70 ± 0.56 |
| scFv-sGFP-2xNLS-Cul1/SKP1-mTagBFP2 | 2.88 ± 0.62 |
| scFv-sGFP-2xNLS-Cul1/RBX1-mCherry/SKP1-mTagBFP2 | 1.67 ± 0.56 |
| scFv-sfGFP-GNG2/GNB1-mCherry | 1.31 ± 0.29 |
| scFv-sfGFP-GNG2/GNB2-mTagBFP2 | 2.50 ± 0.80 |
| GNB1-mCherry/ GNB2-mTagBFP2 | 1.47 ± 0.39 |
| scFv-sfGFP-GNG2/GNB1-mTagBFP2 | 1.52 ± 0.25 |
| scFv-sfGFP-GNG2/GNB2-mCherry | 2.85 ± 0.67 |
| GNB2-mCherry/ GNB1-mTagBFP2 | 1.87 ± 0.25 |
| scFv-sfGFP-1xNLS-CUL1/RBX1-mCherry | 1.43 ± 0.18 |
| scFv-sfGFP-1xNLS-CUL1/SKP1- mTagBFP2 | 1.70 ± 0.56 |
| scFv-sfGFP-CUL1-2xGCN/RBX1-mCherry | 2.06 ± 0.54 |
| scFv-sfGFP-CUL1-10xGCN/RBX1-mCherry | 1.70 ± 0.56 |
| scFv-sfGFP-CUL1-24xGCN/RBX1-mCherry | 1.85 ± 0.41 |
| scFv-sfGFP-CUL1-153xRepeat/RBX1-mCherry | 8.32 ± 2.59 |
| scFv-sfGFP-CUL1-153x-42xRepeat/RBX1-mCherry | 17.64 ± 3.87 |
| scFv-sfGFP-2xNLS-CUL1/CSN2-mCherry | 3.11 ± 0.54 |
| scFv-sGFP-GNG2/GNB1-mCherry | 1.63 ± 0.22 |
| scFv-sGFP-GNG2/GNAi1-mTagBFP2 | 3.42 ± 0.35 |
| GNB1-mCherry/GNAi1-mTagBFP2 | 2.13 ± 0.36 |
| scFv-sGFP-GNG2/GNAQ-mCherry | 4.45 ± 0.51 |
| scFv-sGFP-GNG2/GNB2-mTagBFP2 | 91.67 ± 1.16 |
| scFv-sfGFP-2xNLS-CUL1/NEDD8- mCherry | 3.00 ± 0.41 |
| scFv-sfGFP-2xNLS-CUL1/NEDD8- mCherry&RBX1- mTagBFP2 | 2.90 ± 0.40 |
| SsPB-SKP1-mCherry/GNAI1-mTagBFP2 | –1.29 ± 0.62 |
| NOS1N condensates/PSD95-mCherry | 1.42 ± 0.19 |
| GRB2 condensates/SOS1-mCherry | 1.46 ± 0.19 |

**Supplementary Table 4. Comparative evaluation of major methods for visualizing PPIs in live cells.** **This table** **summarizes the applicability, sensitivity, and specificity of four widely used or emerging platforms:** BiFC, FRET, F3H and MRC. The comparison covers key experimental parameters including compatibility with live-cell imaging, ability to analyze multiprotein complexes and phase separation, sensitivity to transient interactions, risk of false positives, and suitability across different cell types.

| Method | PPI Visualization | Live-cell Compatible | Analyzes Multiprotein Complexes | Analyzes Phase Separation | Sensitive to Transient Interactions | False Positive Risk | Spatial Resolution Demand | Applicable to Multiple Cell Types |
| --- | --- | --- | --- | --- | --- | --- | --- | --- |
| BiFC | Yes | Yes | No | No | No | High | Low | High |
| FRET | Yes | Yes | Limited | No | Yes | Medium | Low | High |
| F3H | Yes | Yes | Unknown | Unknown | Yes | Relatively low | Low | Low |
| MRC | Yes | Yes | Yes | Yes | Yes | Relatively low | Low | High (In theory) |

**Supplementary Table 5.** Comparative analysis of methods for visualizing PPIs in live cells, highlighting key advantages and disadvantages. This table summarizes the core strengths and limitations of four representative techniques: BiFC, FRET, F3H, and MRC for detecting PPIs in live cell contexts.

| Method | Advantages | Disadvantages | Applicable to Multiprotein Interactions | Applicable to Condensate/LLPS Studies | Remarks |
| --- | --- | --- | --- | --- | --- |
| BiFC (Bimolecular Fluorescence Complementation) | Visualizes protein–protein interactions; simple design; strong signal | May produce false positives due to spontaneous complementation; limited to stable interactions; poor detection of transient or weak interactions; irreversible signal; slow fluorophore maturation | Limited applicability | No, not suitable for phase separation studies | Suitable for verifying stable binary interactions |
| FRET (Fluorescence Resonance Energy Transfer) | Capable of detecting transient and dynamic interactions; suitable for real-time imaging | Sensitive to background noise; highly dependent on distance and orientation; complex experimental design; technically demanding | Limited applicability | No, not suitable for studying large complexes or condensates | Requires careful optimization of fluorophore intensity and conformational sensitivity |
| F3H (Fluorescent Three-Hybrid) | Enables spatiotemporal imaging of dynamic interactions; allows real-time monitoring of protein association and dissociation | Restricted to intranuclear interactions; lacks subcellular localization flexibility; requires genomic modification; less adaptable to other types of cells | Unknown | Unknown | Requires genomic integration of Lac operator sequences |
| MRC (Molecular Recruitment Colocalization) | Enables visualization of multiprotein complex formation; suitable for phase separation analysis; supports domain function mapping; programmable, modular, and highly scalable; compatible with optogenetics, quantum dots, etc.; stable signals for live-cell imaging | Currently limited to nuclear interactions; lacks subcellular localization flexibility | Yes, applied to multi-protein complexes | Yes, applicable for identifying, inducing, and manipulating phase separation and multiphase condensates | Compatible with SunTag, sgRNA, optogenetics tool, and single-molecule tracking platforms; offers broad application potential |

**Supplementary Table 6. Troubleshooting guide for adapting the MRC system.** This table summarizes common issues encountered when using the MRC system, along with potential causes and recommended optimization strategies. It is intended to help users rapidly identify and resolve technical problems when testing different protein combinations in live-cell recruitment and colocalization assays.

| Observed Problem | Possible Cause | Optimization Strategy |
| --- | --- | --- |
| Weak or low SNR signal at targeted loci | Insufficient sgRNA repeat number / weak dCas9 recruitment | Increase sgRNA target repeats (e.g., add 42 repeats), verify sgRNA efficiency |
| High background / non-specific nuclear spots | Too many GCN4 repeats / overexpression | Reduce GCN4 repeat number (e.g., from 24x to 10x), use weaker promoters |
| Signal is visible but no colocalization observed | Proteins do not interact / require third party | Test known interactors, add bridging protein (e.g., CUL1 for SKP1–RBX1 interaction) |
| Low reproducibility of colocalized signals | Variability in transfection / expression imbalance | Normalize transfection ratios, use stable cell lines, co-expression from single vector |
| Excessive nuclear dots (>3 per cell) | Over-recruitment / off-target dCas9 binding | Use optimal GCN4 repeat number (e.g., 10x), optimize sgRNA specificity |
| Weak or absent signal from large proteins (e.g., CUL1) | Cytoplasmic retention / poor nuclear import | Add 2xNLS, truncate non-essential domains if possible |

**Supplementary Table 7.** **Mobile fraction and recovery time constant for different synaptic proteins measured by FRAP. FRAP analysis was performed to evaluate the dynamics of various synaptic scaffold proteins.** The mobile fraction (A) represents the proportion of fluorescent molecules able to move back into the bleached region, while the recovery time constant (τ) reflects the rate of fluorescence recovery. Percentage recovery was derived from the fitted mobile fraction. Values are expressed as mean ± SD from at least three independent measurements.

| Protein | Mobile Fraction (A) |  | % Recovery |
| --- | --- | --- | --- |
| NOS1N | 0.551 ± 0.180 |  | 55.11 ± 18.02 |
| APBA | 0.136 ± 0.021 |  | 13.55 ± 2.10 |
| GRIP | 0.272 ± 0.036 |  | 27.20 ± 3.63 |
| MPDZ | 0.281 ± 0.036 |  | 28.15 ± 3.56 |
| TJP1 | 0.056 ± 0.020 |  | 5.58 ± 1.97 |
| PSD95 | 0.111 ± 0.015 |  | 11.14 ± 1.55 |

**Supplementary Movie 1: Single-molecule tracking NOS1N-NLS-BAP-QDs inside NOS1N condensates in Living Cells using MRC, related to Fig. 9B and C.** Single-particle tracking was performed at a frame rate of 100 ms andotal tracking time was 10 s. The movie's frame rate is 20 frames per second. Scale bar, 0.5μm.

**Supplementary** **Movie 2: The 561 nm channel of Movie S1. Individually track the trajectories of QDs.**
